# Imaging local brain activity of multiple freely moving mice sharing the same environment

**DOI:** 10.1038/s41598-019-43897-x

**Published:** 2019-05-16

**Authors:** Shigenori Inagaki, Masakazu Agetsuma, Shinya Ohara, Toshio Iijima, Hideo Yokota, Tetsuichi Wazawa, Yoshiyuki Arai, Takeharu Nagai

**Affiliations:** 1 Graduate School of Frontier Biosciences, Osaka University, Suita Osaka, 565-0871 Japan; 2 The Institute of Scientific and Industrial Research, Osaka University, Ibaraki Osaka, 567-0047 Japan; 3 Division of Systems Neuroscience, Tohoku University Graduate School of Life Sciences, Sendai, Miyagi 980-8577 Japan; 4 Image Processing Research Team, RIKEN Centre for Advanced Photonics, Wako Saitama, 351-0198 Japan; 5 Present Address: Graduate School of Medical Sciences, Kyushu University, Fukuoka, 812-8582 Japan; 6 Present Address: Division of Homeostatic Development, Department of Developmental Physiology, National Institute for Physiological Sciences, Okazaki, Aichi 444-8585 Japan

**Keywords:** Bioluminescence imaging, Visual system

## Abstract

Electrophysiological field potential dynamics have been widely used to investigate brain functions and related psychiatric disorders. Considering recent demand for its applicability to freely moving subjects, especially for animals in a group and socially interacting with each other, here we propose a new method based on a bioluminescent voltage indicator LOTUS-V. Using our fiber-free recording method based on the LOTUS-V, we succeeded in capturing dynamic change of brain activity in freely moving mice. Because LOTUS-V is the ratiometric indicator, motion and head-angle artifacts were not significantly detected. Taking advantage of our method as a fiber-free system, we further succeeded in simultaneously recording from multiple independently-locomotive mice that were freely interacting with one another. Importantly, this enabled us to find that the primary visual cortex, a center of visual processing, was activated during the interaction of mice. This methodology may further facilitate a wide range of studies in neurobiology and psychiatry.

## Introduction

Brain functions and related psychiatric disorders have been investigated by recording electrophysiological field potentials^1^. Electroencephalography (EEG) measures whole brain wide activity, while local field potential recording detects the dynamics of more localized micro-networks of the neurons^2^. More recently, the development of fluorescence-based organic dyes and genetically encoded indicators to detect neuronal activity has proven useful for brain-wide and local micro-network level recordings^1,3–9^. Both electrophysiological and fluorescence-based techniques have been further developed to record freely moving animals, via electrical or optic fibers connected to them. This allows the detection of brain activity during more complicated behavioral tasks^1,10–13^.

However, there are still several technical limitations associated with the use of these existing methods. One limitation is that the subject needs to be head-fixed or connected to the cable (electrical or optical) in most cases, which limits the application of these techniques to freely moving subjects. Although there are several ways to apply those techniques to the recording during social interaction of animals^14–17^, most of the recordings were performed only from either a single animal or animals with restricted interaction in order to avoid tangling of recording cables. Therefore, simultaneous recording from a group of multiple animals freely interacting with each other has still been challenging. Wireless devices for the neural activity recording have also been developed^18^ and the miniaturized types was developed recently^19,20^. However, they are still more or less invasive and have not been published to be applicable for simultaneous recording of multiple mice freely interacting with each other.

Bioluminescent probes, which don’t require excitation light for the signal emission, have been demonstrated useful to monitor the signals in freely moving animals^21,22^. It has also been shown applicable to non-invasively detect protein expression level in the brain^23,24^. Importantly, since those imaging methods were performed without recording cables, bioluminescence imaging can potentially be a powerful method for the simultaneous recording of brain activities from multiple animals during social interaction. However, neither bioluminescence-based *in vivo “*live” detection of dynamically changing brain activity during free moving, nor bioluminescence imaging simultaneously from multiple animals has been reported.

Here we report a novel fiber-free recording method using a recently described bioluminescence-based voltage indicator “LOTUS-V”^25^. Our method enables simultaneous recording from multiple mice freely interacting with each other, using a simple optical setup. Since LOTUS-V is a ratiometric indicator, we detected no significant artifact caused by mouse’s locomotion and head angle. Using this method, we actually detected neural activity related to social behaviors, suggesting its further potential applicability to a wide range of studies in neurobiology and psychiatry.

## Results

### Voltage imaging in neurons using LOTUS-V

LOTUS-V consists of a voltage-sensing domain (VSD) from voltage-sensing phosphatase (Ci-VSP)^26^, NLuc (i.e., a cyan-emitting luciferase that is approximately 150 times brighter than Renilla or firefly luciferases)^27^, and Venus (i.e., a yellow fluorescent protein)^28^. An increase of the membrane voltage causes a structural change of the VSD, enhancing Förster resonance energy transfer (FRET) between the NLuc and Venus, which consequently decreases the NLuc signal and increases the Venus signal and thus allows the ratiometric measurement (Supplementary Fig. 1). We previously demonstrated that the ratiometric measurement by LOTUS-V is useful to monitor voltage changes in HEK293T cultured single cells and moving cardiomyocyte aggregates. We verified the advantages of LOTUS-V in long-term imaging and the robustness of the signal reliability in a moving specimen^25^.

**Figure 1:**
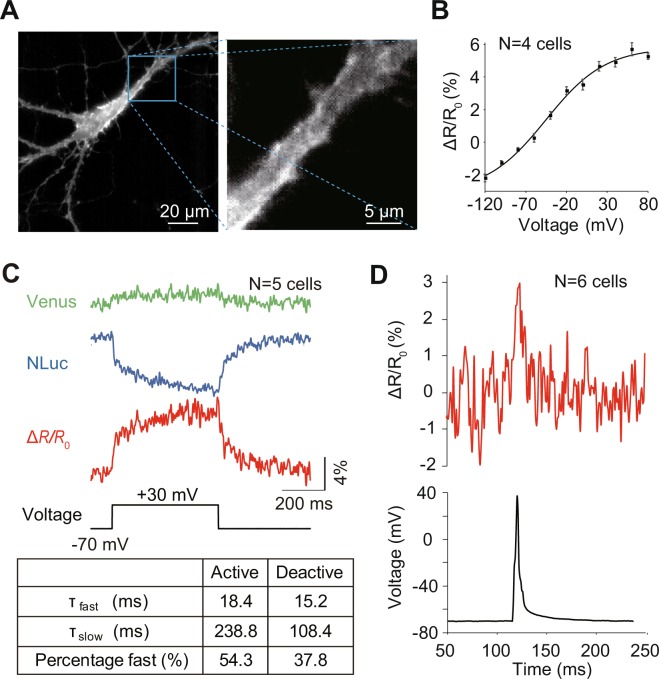
Electrophysiological characterization of LOTUS-V in hippocampal neurons. (**A**; left) A representative bioluminescence image of a cultured hippocampal neuron expressing LOTUS-V. (**A**; right) An expanded image of the region within the square on the image on the left. (**B**) Plot of fractional Δ*R*/*R*_0_ versus voltage changes (n = 4 cells). The Δ*R*/*R*_0_ from −120 mV to + 80 mV was 5.3 ± 0.3%. The effective valence (Z) was 0.7, while the V_1/2_ was −45.5 mV. The plot was fitted using a Boltzmann function. (**C**) The Venus and NLuc signals (Δ*L*/*L*_0_), and their ratio (Δ*R*/*R*_0_), in response to voltage changes from the holding voltage (−70 mV to + 30 mV; n = 5 cells). (**C**; table) The fast and slow components, and their fraction of time constant. The activation and deactivation curves of Δ*R*/*R*_0_ were fitted using a two-component exponential equation. (**D**; upper) Action potential waveform of Δ*R*/*R*_0_ and (**D**; lower) electrophysiology (n = 6 cells). The imaging frame rate was 1 kHz. Error bars indicate mean ± standard error.

To validate the efficacy of LOTUS-V in voltage imaging of neurons, we first expressed it in primary cultures of rat hippocampal neurons, and investigated signal changes during patch clamp recording (Fig. 1 and Supplementary Fig. 2). An intense bioluminescence was observed from a single neuron on addition of furimazine, a substrate for the NLuc moiety in LOTUS-V (Fig. 1A and Supplementary Fig. 1B). Thus we could record the signal using a 1-kHz frame rate (Fig. 1C,D and Supplementary Fig. 2). As previously observed in HEK293T cells^25^, LOTUS-V had wide-range detectability of voltage change in neurons (Fig. 1B; −120 mV to +80 mV). The time constants of the voltage response (Fig. 1C) were like those of a conventional fluorescent indicator, ArclightQ239^29,30^. During neuronal firing, a significant signal increase of LOTUS-V was observed (Fig. 1D; baseline-to-peak amplitude, 2.80 ± 0.88% [mean ± SE]; n = 6 cells; p < 0.01, paired t-test). In addition, the calculation of the emission intensity ratio from two channels rather than a single channel was advantageous to obtaining a larger signal change (Supplementary Fig. 2A). Even a single trial or averaging a small number of trials could produce a clear signal change (Supplementary Fig. 2B). The majority of recorded cells independently showed significant signal changes (Supplementary Fig. 2C). These results substantially confirmed the efficacy of LOTUS-V in voltage imaging of neurons, which further suggested that it could reliably report *in vivo* brain activity (i.e., electrophysiological field potential dynamics derived from neuronal population). A previous study also demonstrated that LOTUS-V was advantageous to analyze voltage changes in a mass of cells^25^.

### LOTUS-V can report brain activity in an awake and head-fixed mouse

**Figure 2:**
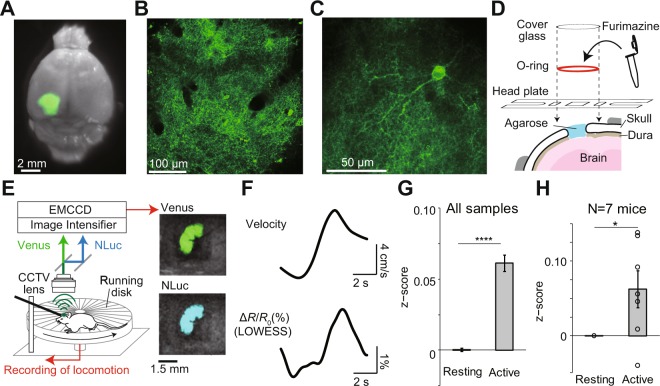
Imaging of a head-fixed mouse with LOTUS-V. (**A**) Venus fluorescence in the primary visual cortex (V1) of a paraformaldehyde-fixed brain. (**B**,**C**) Examples of two-photon fluorescence images of V1 area containing LOTUS-V-expressing neurons (**B**) and a single V1 neuron spatially well resolved by two-photon microscopy (**C**). (**D**) Schematic drawing of the prepared cranial window. The headplate and o-ring were glued over the cranial window. The furimazine solution was enclosed in the o-ring bath by a cover glass. (**E**; left) Schematic drawing of the imaging setup for a head-fixed mouse. A mouse was placed on a running disk while the head was fixed to a bar via the headplate. The velocity was recorded through a rotary encoder during imaging. LOTUS-V bioluminescence from the V1 was collected through a CCTV lens. NLuc and Venus emissions were separated by image splitting optics. These emissions were acquired using an image intensifier unit, which can amplify the signal up to 10^6^ times, and recorded with an EMCCD camera in the dark condition. (**E**; right) Overlaid images of bright-field and bioluminescence in NLuc and Venus channels acquired by this system. (**F**) Averaged time series of velocity and LOWESS-smoothed Δ*R*/*R*_0_ at the locomotion onset (n = 12 sessions from N = 3 mice). The Granger causality test was performed to determine whether the velocity Granger-causes Δ*R*/*R*_0_ statistically (p < 0.05). (**G**,**H**) Plots of z-normalized Δ*R*/*R*_0_ in resting (<5 cm/s) and active (>5 cm/s) states of head-fixed mice, using (**G**) all time-points (p < 0.0001, Wilcoxon rank sum test; n = 707871; 31653 time-points), or (**H**) average values from each mouse (p < 0.05, Wilcoxon signed-rank test; N = 7 mice). Error bars indicate mean ± standard error; ^*^p < 0.05; ^****^p < 0.0001.

We first tested the efficacy of LOTUS-V for *in vivo* imaging of brain activity from an awake mouse, using a head-fixed system. LOTUS-V was locally expressed in the primary visual cortex (V1) using the adeno-associated virus (AAV) gene expression system, and labelled a population of local neurons in the V1 (Fig. 2A–C). Since it was already well confirmed that neural activity in the V1 was correlated with the locomotion of mice, even in the absence of visual input or in the dark^31–33^, we tested whether LOTUS-V signal could reflect such a locomotion-dependent increase of neural activity.

The ratio change of the LOTUS-V signal in the V1 of head-fixed mice was recorded (Fig. 2D–H), simultaneously with the speed of their spontaneous locomotion using the rotation of a running disk placed under the mouse (Fig. 2E). Using this system, we observed the enhanced signal when mice were locomoting on the running disk (Fig. 2F–H) and found that the locomotion possessed significant Granger causality to the LOTUS-V signal (p < 0.05, the Granger causality test) (Fig. 2F), suggesting that the temporal resolution of our method is substantially helpful to investigate causality between behaviors and brain activity.

Since V1 neurons strongly respond to visual stimulation^3,6,8^, we also investigated the signal change of the LOTUS-V during visual stimulation, to further elucidate its applicability for variable modalities (Supplementary Fig. 3). Light illumination for the visual stimulation can be reflected and contaminated in the camera, consequently masking the images. Therefore, we conducted the “dead-time imaging”^25,34^, where the exposure to the camera and visual stimulation during the processes for image readout and accumulated charge clearing on the camera (dead-time) were alternately (not simultaneously) performed (Supplementary Fig. 3A,B). LOTUS-V successfully reported an increase in signal, depending on the light intensities (Supplementary Fig. 3C), which confirmed its applicability to monitor changes in local brain activity, irrespective of the input modality.

### LOTUS-V can report brain activity from a freely moving mouse

**Figure 3:**
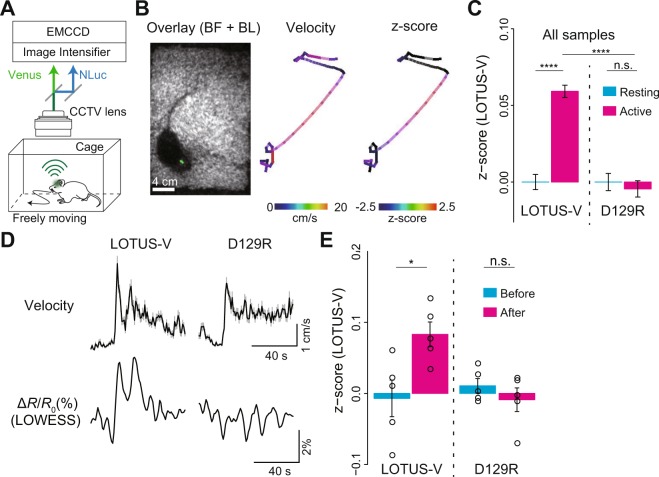
Imaging of V1 activity in a freely moving mouse using LOTUS-V and an automatic tracking system. (**A**) Schematic diagram of imaging of a freely moving mouse. The mouse was placed in its home cage and the LOTUS-V bioluminescence was recorded. (**B**; left) Overlaid image of bright field and LOTUS-V bioluminescence (green). (**B**; middle and right) Pseudo-colored trajectories of mouse locomotion, indicating velocity (middle) and z-normalized Δ*R*/*R*_0_ (right) (see also Supplementary Videos 1 and 2). (**C**) Bar plots of z-normalized Δ*R*/*R*_0_ in the resting (<1 cm/s) and active (>1 cm/s) states of freely moving mice (p < 0.0001 for Kruskal-Wallis test with all four categories; resting and active states of LOTUS-V, n = 41005 and 69826 time-points from N = 5 mice; resting and active states of LOTUS-V(D129R), n = 31648 and 34006 from N = 3; p-values shown in the panel were calculated using a Steel-Dwass test). (**D**) Averaged time series of velocity and LOWESS-smoothed Δ*R*/*R*_0_ at the locomotion onset (LOTUS-V, n = 29 sessions from N = 5 mice; D129R, n = 39 sessions from N = 3 mice). The Granger causality test was applied to determine whether the velocity Granger-causes Δ*R*/*R*_0_ (p < 0.01 and n.s. for LOTUS-V and LOTUS-V(D129R), respectively) (**E**) The z-normalized Δ*R*/*R*_0_ before (−19 s to 0 s) and after (1 s to 20 s) the locomotion onset. While the circles indicate z-normalized Δ*R*/*R*_0_ at each time point, the bar plots show the average in each category. P-value shown in the panel was calculated using the Wilcoxon rank sum test. Time bin, 0.1 s (**B**,**C**), 1 s (**D**) and 4 s (**E**,**F**); Error bars indicate mean ± standard error; n.s., not significant; ^*^p < 0.05; ^****^p < 0.0001.

Next, we examined whether LOTUS-V can report brain activity in a freely moving mouse. Conventionally, brain activity imaging from a freely moving mouse requires multiple optical fibers to excite and detect fluorescent voltage or calcium indicators, and an additional infrared video camera to detect locomotion^1,11^. In contrast, our method only required a mouse to be placed in the cage, with a completely detached CCD camera system fixed above the mouse cage (Fig. 3A). During the experiments, a strong bioluminescence from the targeted brain region was continuously observed for 1.15–6.77 h (3.10 ± 0.45 h [mean ± SE]; N = 16 mice), enabling us to perform long-term imaging. Considering TEMPO^1^, i.e., a method recently developed for fiber-coupling based voltage imaging, has been reported to enable observation for approximately 1 h, our method may be more advantageous for long-term observation. Moreover, since our method does not require illumination light, limitations due to laser fluctuation, auto-fluorescence, and bleaching do not have to be considered. The recording in the present study was performed without inserting any devices into the brain, which is unavoidable when using many of other methods for the brain activity recording, suggesting that our method can be less invasive.

The position change of the bioluminescent spot in a freely moving mouse was detected using a particle track analysis program (Fig. 3B, Supplementary Fig. 4 and Supplementary Video 1). We could automatically detect the LOTUS-V signal in the V1 and velocity (Fig. 3B and Supplementary Video 2). The z-score (normalized values calculated from Δ*R*/*R*_0_, which has been used to summarize the results from different mice^35^) increased significantly during the active state of LOTUS-V expressing mice (Fig. 3C; p < 0.0001, Wilcoxon rank sum test), in corroboration with previous studies^31–33^. To evaluate whether this signal change reflected a real voltage change or was just an artifact, we compared the results of LOTUS-V expressing mice (N = 5 mice) with those expressing voltage-insensitive LOTUS-V(D129R) (N = 3 mice)^25,36^. Locomotion did not significantly change the LOTUS-V(D129R) signals (Fig. 3C). Further, the LOTUS-V signal during the active state was statistically higher than the LOTUS-V(D129R) signal during the active state (Fig. 3C; p < 0.0001, Kruskal-Wallis one way analysis of variance for all categories; p < 0.0001 for the resting vs active states of LOTUS-V, n.s. for the resting vs active states of LOTUS-V(D129R), p < 0.0001 for LOTUS-V during the active state vs LOTUS-V(D129R) during the active state, Steel-Dwass multiple comparison test).

To consider the existence of motion-driven artifact more precisely, we compared the locomotion speed and the signal of LOTUS-V or LOTUS-V(D129R) (Supplementary Fig. 5). The LOTUS-V signal increased depending on the locomotion speed (Supplementary Fig. 5A,C). On the other hand, LOTUS-V(D129R) signal showed no significant trend of signal change (Supplementary Fig. 5A,D). We also statistically considered the possibility that the samples from each mouse were correlated by a linear mixed-effect model and obtained a similar result (Supplementary Fig. 5B). In addition, the LOTUS-V signal increased by the locomotion was significantly higher than the LOTUS-V(D129R) signal at the same locomotion speed (Supplementary Fig. 5A,B). Therefore, these results support that the increase in LOTUS-V signal, which we observed due to the mouse locomotion, was not caused by the motion artifact. We further tested whether the changes in head angle during the free moving might induce artifacts in the recording of brain activity. Although each signal (NLuc and Venus) recorded independently decreased according to the tilt of the mouse head (Supplementary Fig. 6), we confirmed that the calculated ratio of the signals, which we used for the analyses of the LOTUS-V signal, was not significantly affected by the head angle (Supplementary Fig. 6B). The analysis based on a linear mixed-effect model also indicated that the difference between the ratio values was not significantly affected by the head angle (Resting, p = 0.553; Active, p = 0.053; Resting and Active, p = 0.160, with ANOVA for a linear mixed-effect model). Subsequently, we tested whether vertical motion affected the LOTUS-V signal. While the vertical position of mouse’s head changed during grooming and standing-up, the ratio values between different phases were not significantly different (Supplementary Fig. 7). These results suggest that the ratiometry could mitigate the various artifacts and enable robust and reliable detectability of the brain activity.

To evaluate the temporal precision of the LOTUS-V signal, we checked the time course change of LOTUS-V signal before and after the locomotion onset (Fig. 3D,E, Supplementary Figs 8 and 9). We found that the LOTUS-V signal changed more or less similarly to the locomotion change (Fig. 3D and Supplementary Fig. 8), and significantly increased after the locomotion onset (Fig. 3E and Supplementary Fig. 9), while LOTUS-V(D129R) signal did not significantly change (Fig. 3D,E), revealing the reliability of the LOTUS-V signal (LOTUS-V, Δ*R*/*R*_0_ = −0.19 ± 0.62% and 1.87 ± 0.44% for “Before” and “After” the onset, respectively; p < 0.05, Wilcoxon rank sum test, n = 5 time-points (−19 s to 0 s and 1 s to 20 s, respectively); D129R, Δ*R*/*R*_0_ = 0.28 ± 0.25%; and −0.24 ± 0.42%, n.s., n = 5). Furthermore, we found that the mouse locomotion possessed significant Granger causality to the LOTUS-V signal (Fig. 3D; p < 0.01 and n.s. for LOTUS-V and LOTUS-V(D129R), respectively, the Granger causality test) (see also Supplementary Fig. 8), suggesting that the increase of the LOTUS-V signal likely reported the change in the brain activity triggered by the increased locomotion. These results suggest that our method allows to investigate temporal dynamics of brain activity triggered by specific behavioral events. A previous study^37^ reported that locomotion-driven change in the neural activity of V1 is mild in the dark or when visual stimulation is absent. It was, however, significantly detected using our system, suggesting the substantial sensitivity of our method for imaging a freely moving mouse.

### LOTUS-V can report brain activity from interactively moving mice

**Figure 4:**
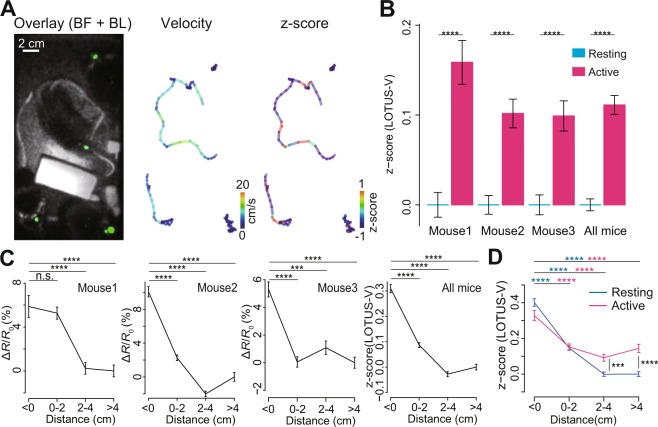
Imaging of interactively moving mice. (**A**; left) Overlaid image of bright-field and LOTUS-V bioluminescence from multiple mice (green). (**A**; middle and right) Pseudo-colored locomotion trajectory, indicating velocity (middle) and z-normalized Δ*R*/*R*_0_ from each mouse (right) (see also Supplementary Videos 3 and 4). (**B**) Bar plots of z-normalized Δ*R*/*R*_0_ during resting (<1 cm/s) and active (>1 cm/s) states of freely-moving multiple mice, demonstrating locomotion-dependent signal increases (Mouse 1, n = 5307 and 2066 time-points; Mouse 2, n = 9192 and 4721; Mouse 3, n = 8100 and 4385; All mice, n = 22599 and 11172). P-values were obtained using the Wilcoxon rank sum test. (**C**) Distance-dependent change in the activity of the primary visual cortex (V1) of interactively locomoting mice. Plots represent Δ*R*/*R*_0_ or z-normalized Δ*R*/*R*_0_ of each distance category (distance between the target mouse nose and other mice, see also (Supplementary Fig. 10; distance from Mouse 1, n = 688, 2338, 2118, and 2229 time-points for < 0, 0–2, 2–4 and > 4 cm, respectively; Mouse 2, n = 2239, 5382, 4226, and 2066; Mouse 3, n = 2516, 4019, 2313, and 3637). The distant values (>4 cm) were used as a baseline (*R*_0_) to calculate the Δ*R*/*R*_0_ (for Mouse 1–3) and for the z-normalization (for “all mice”). p < 0.0001 for Kruskal-Wallis test with all four categories. P-values shown in the panel were calculated using a Steel-Dwass test. (**D**) Comparison of distance-dependent change in V1 activity and the effect of locomotion (resting vs active states). Data from all mice were used (resting state (<1 cm/s, blue), n = 3538, 7606, 5843, and 5612 time-points for <0, 0–2, 2–4, and >4 cm, respectively; active state (>1 cm/s, magenta), n = 1905, 4133, 2814, and 2320). The Δ*R*/*R*_0_ in the “distant and resting” state was used as the baseline for z-normalization. P-values obtained using Kruskal-Wallis test with all four categories; p < 0.0001 in both states. P-values obtained using Steel-Dwass test are shown as blue (resting states) or magenta (active) symbols, while those obtained using the Wilcoxon rank sum test (to compare resting vs active) are shown in black. The time bin was 0.1 s. Error bars indicate mean ± standard error; n.s., not significant; ^***^p < 0.001; ^****^p < 0.0001.

Finally, we tested whether our fiber-free method could enable simultaneous recording of multiple mice that were freely interacting with each other (Fig. 4 and Supplementary Video 3). To clearly capture the mouse body during imaging of LOTUS-V signal in the brain, the inside of the cage was illuminated with a light emitting diode, and bright-field and bioluminescence images were taken alternately^21,22^. Thereafter, we could easily track the target areas (multiple part of the body) in each mouse, and simultaneously measured the locomotion and LOTUS-V signal. Using this method, apart from a mouse that was quiet throughout the imaging and thus excluded from the analysis (see Supplementary methods), the locomotion-driven signal change in the V1 of three other freely interacting mice was successfully detected (Fig. 4A,B). Although some wireless technology for EEG recording has been reported to enable detection of whole brain wide activity (http://www.vyssotski.ch/index.html), which may further be applicable for recording from multiple mice, our work is the first report of simultaneous recording of “specifically-targeted” brain regions from freely-interacting multiple mice. Further, since this experiment was performed under the blinking light as described above, it demonstrated that brain activity recording based on bioluminescence can not only be performed in the dark, but also under the semi-illuminated situation where animals can perform visually-guided behaviors and cognition.

More importantly, by systematically analyzing the distance between the mice (Supplementary Fig. 10), we found that V1 activity was significantly higher when each mouse approached the others (Fig. 4C). In addition, by separately analyzing the locomotion-dependent and distance-dependent effects (Supplementary Fig. 11), we demonstrated that locomotion did not significantly increase brain activity in the V1 when the distance was small (<2 cm) (Fig. 4D). We also investigated the result via a linear mixed-effect model and confirmed that a similar result was obtained (Supplementary Fig. 12). This suggested that the interaction with other mice may have a stronger or more competitive impact on the V1 compared to simple exploratory locomotion.

Altogether, these results indicated that brain activity imaging with LOTUS-V can be a powerful method to uncover novel brain function during social interaction. One concern was that the distance dependent increase of the signal might happen simply because of leaky signals emitted from other nearby mice. If this was the case, signals of two mice should affect each other at each moment when their distance got closer, which should result in positive correlation of the signals between mice. To test this possibility, we calculated the correlation coefficients between the LOTUS-V signals from pairs of mice, and checked the distance dependent artifacts. As a result, we found that correlation coefficients did not increase by the decrease of the distances between mice (Supplementary Figs 13–16). This result suggests that the distance dependent increase of V1 activity is unlikely due to the leaky signals from other mice, supporting the reliability of our method to measure change in the brain activity simultaneously from multiple animals during their free interaction.

## Discussion

Present study demonstrated that imaging with the LOTUS-V is a powerful and motion-artifact-free method to monitor neural activity from a target brain area of freely moving multiple mice. To our knowledge, while several reports previously showed that bioluminescent proteins can report signals from freely moving animals^21–24^, this is the first report demonstrating the live imaging technique to detect brain activity dynamics using a bioluminescent probe in a freely moving animal. For example, while one of the previous studies demonstrated that a red-shifted luciferase was useful to detect the bioluminescent signal in the brain of freely moving animals^23^, they only demonstrated the detection of signals from neural populations by the reporter system based on the c-fos promoter (i.e., temporal resolution is low: they needed to wait ~7 hours after neurons were activated for the expression of the reporter bioluminescent protein under the regulation of the c-fos promoter, and >4 days until the expression level returned to baseline levels). In contrast, we could demonstrate seconds-order temporal resolution *in vivo* (Figs 2F and 3D). Although the temporal resolution is still inferior to the fiber-based method, further improvement of the voltage indicator should enhance the usefulness of our method in broader research fields, as discussed below.

Our method is less invasive comparing with the method based on the insertion of an optical or electrode fiber. More importantly, this fiber-free imaging method enabled us to simultaneously record the brain activities from interactively moving mice, and demonstrated a novel type of V1 activation during the interaction.

Since the present method uses a genetically encoded indicator, our method is applicable for tissue or cell-type-specific recording, as well as whole-brain macroscopic imaging, all of which are widely required in neuroscience research^38^. Furthermore, since optogenetic manipulation is compatible with LOTUS-V imaging as we previously reported^25^, a wireless optogenetics device^39,40^, which could be combined to our fiber-free imaging method, may allow us to wirelessly detect and manipulate neural activity in socially interacting animals.

Although we demonstrated that our method has a strong potential to open new research fields in neuroscience, further improvements and tests are important to encourage the practical usefulness of this technique. First, improvement of the signal-to-noise ratio will be a key for enhancement of temporal resolution and reliability of the detected signals. Since the photon number emitted from a luciferase is generally 100–1000 times lower than that from fluorescent proteins, we introduced the image intensifier to enhance the signals to maintain high temporal resolution. However, when the gain of the image intensifier increased, a dark noise was also enhanced, which decreased the signal-to-noise ratio. Therefore, the development of a brighter luciferase is expected to solve this issue. It is important to note that we confirmed that the bioluminescent indicator should be FRET-based for the quantitative measurement of brain activity in freely moving animals (Supplementary Fig. 6), and such a new luciferase therefore should be suitable to design a FRET-based indicator. Second, it would be interesting to test the applicability of our method to the recording in a deep brain region, and further improvement for this purpose is also important. While we demonstrated the usefulness of our method to detect the activity of the superficial layer of the V1 (~300 μm depth), investigation of a deeper brain region is also important in general. Insertion of GRIN lens is a popular surgery procedure to access the deep brain regions currently, which will be helpful to improve the accessibility to the deeper brain regions (i.e., bioluminescent indicators that we express in the deep target area can theoretically be detected as ones from the brain surface by introducing this method). On the other hand, this procedure is invasive, and thus, may perturb normal brain function. Since a recently reported red-shifted luciferase allows noninvasive detection of bioluminescent signals from a striatum^23^, it could be interesting to challenge to develop a new FRET-based voltage indicator based on this; though it is generally difficult to develop a FRET-based indicator with red-shifted reporter proteins. Third, our method may be applicable for chronic and continuous recordings. We demonstrated ~6 hours of continuous recording, but due to the consumption of the substrate enclosed at the cranial window (Fig. 2D), the following bioluminescent signal became too weak to detect. According to the previous study, the bioluminescent signal should be maintained as long as the fresh substrate is supplied^24^, so it will be important to introduce these techniques and combine them with our method. Fourth, the auto-tracking system should be further improved to detect multiple signals. In the present study, we tracked signals from multiple animals manually to precisely distinguish signals of different animals (Fig. 4). To improve the throughput, on the other hand, a new auto-tracking method to detect multiple signals should be developed. A near infrared fluorescent dye might be applicable to robustly indicate the position of the target and reliably detect the signals from multiple spots^24^. In addition, near infrared light should theoretically enable the detection of the animal’s shape as well, which is generally useful for the automatic detection of animal positions during social interactions^11^. Combining these methods will be helpful to make our auto-tracking strategy further applicable to detect the signals from multiple mice.

Overall, our method opens a door to an easy and minimally invasive recording of brain activity simultaneously in multiple socially interactive animals, thus, contributing to a wide range of neuroscience research.

## Methods

Details are available in the Supplementary methods.

### Animals

All experimental procedures were conducted according to the Institutional Guidance on Animal Experimentation and with permission from the Animal Experiment Committee of Osaka University (authorization number: 3348). C57BL/6JJmsSlc male mice were purchased from Japan SLC, Inc. The mice were housed in the Osaka University Animal Facility and supplied with food and water *ad libitum*.

### Statistical analyses

Statistical analyses were conducted using R software and MATLAB. The type of the statistical analysis conducted for each analysis was described in the manuscript. All p-values less than 0.0001 were described as “P < 0.0001.” All statistical tests, except the Chi-squared test, were performed as “two-tailed” tests. Statistical significance was set at P < 0.05.

## Supplementary information

Supplementary information

Supplementary Video 1.

Supplementary Video 2.

Supplementary Video 3.

Supplementary Video 4.

## Data Availability

The LOTUS-V nucleotide sequence can be accessed in GenBank (accession number; LC061443). LOTUS-V/pcDNA3 is available through Addgene (Plasmid #87127).
