# Supplementary material for: Imaging local brain activity of multiple freely moving mice sharing the same environment
Source: Sci Rep. 2019 May 16;9:7460. doi: 10.1038/s41598-019-43897-x (PMC6522513; doi:10.1038/s41598-019-43897-x)
Supplement: Supplementary file 1 — Supplementary information [file 41598_2019_43897_MOESM1_ESM.pdf]

Imaging local brain activity of multiple freely moving mice sharing the same environment

Shigenori Inagaki[1,5]‡, Masakazu Agetsuma[2,5]§, Shinya Ohara[3], Toshio Iijima[3], Hideo Yokota[4], Tetsuichi Wazawa[2], Yoshiyuki Arai[2] & Takeharu Nagai[1,2]*

[1] Graduate School of Frontier Biosciences, Osaka University, Suita, Osaka 565-0871, Japan

[2] The Institute of Scientific and Industrial Research, Osaka University, Ibaraki, Osaka 567-0047, Japan

[3] Division of Systems Neuroscience, Tohoku University Graduate School of Life Sciences, Sendai, Miyagi 980-8577, Japan

[4] Image Processing Research Team, RIKEN Centre for Advanced Photonics, Wako, Saitama, 351-0198, Japan

[5] These authors contributed equally to this work.

‡ Present address: Graduate School of Medical Sciences, Kyushu University, Fukuoka 812-8582, Japan

§ Present address: Division of Homeostatic Development, Department of Developmental Physiology, National Institute for Physiological Sciences, Okazaki, Aichi 444-8585, Japan

*Correspondence should be addressed to T. N.
ng1@sanken.osaka-u.ac.jp

Tel: +81-6-6879-8481

Fax: +81-6-6875-5724

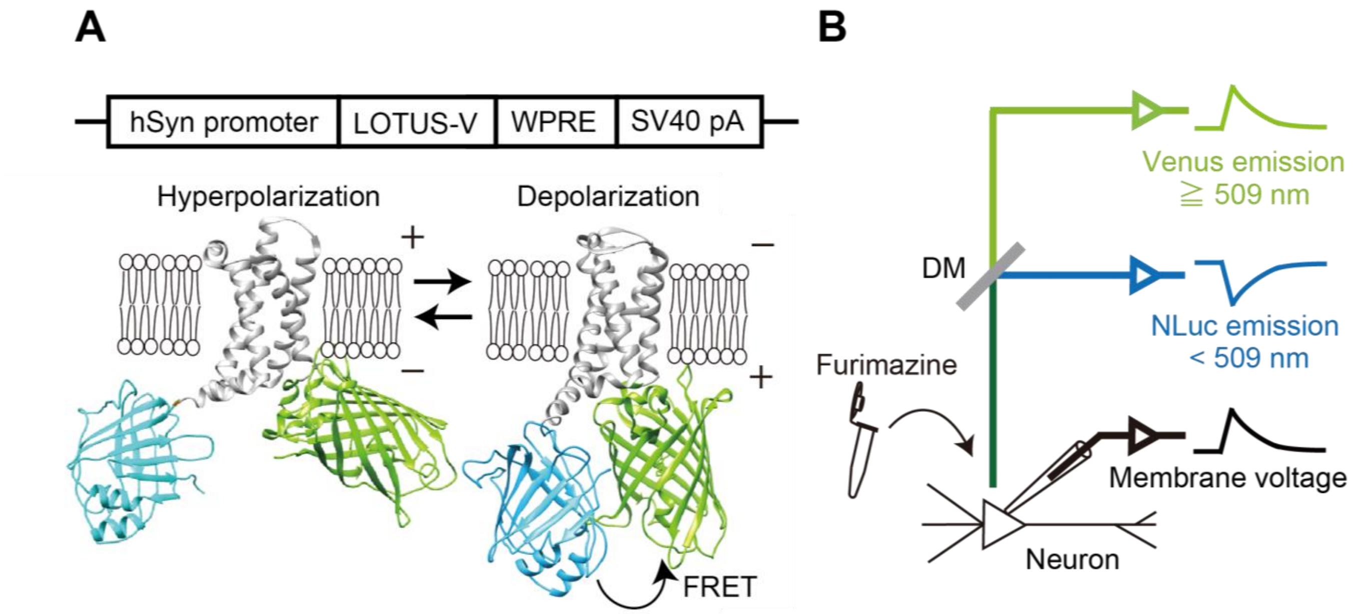

Supplementary Figure 1. Schematic drawing of the LOTUS-V construct and in vitro bioluminescence imaging. (**A**) LOTUS-V was expressed under the control of a hSyn (pan-neuronal) promoter. A woodchuck hepatitis virus post-transcriptional regulatory element (WPRE) and polyadenylation sequence (pA) are located downstream of the LOTUS-V gene to stabilize its mRNA (upper). The LOTUS-V was comprised of VSD (gray), NLuc (cyan), and Venus (yellowish green). The protein structure of VSD (4G7V) and Venus (GFP; 1KYS) were obtained from the Protein Data Bank (PDB), and that of NLuc was predicted by I-TASSER[1]. The structural change upon depolarization enhances Förster resonance energy transfer (FRET) efficiency between NLuc and Venus moieties, and causes a higher emission ratio (Venus/NLuc) (lower). (**B**) Bioluminescence from a cultured hippocampal neuron expressing LOTUS-V was split using a dichroic mirror, and separately recorded at the NLuc and Venus channels (<509 nm and ≥509 nm, respectively). Simultaneously, the LOTUS-V-expressing neuron was subjected to whole-cell patch-clamp recording. While the Venus signal was positively correlated with membrane voltage, the NLuc signal was anti-correlated with it because of the change in FRET efficiency. This emission ratio (Venus/NLuc) can robustly report membrane voltage of cultured hippocampal neurons.

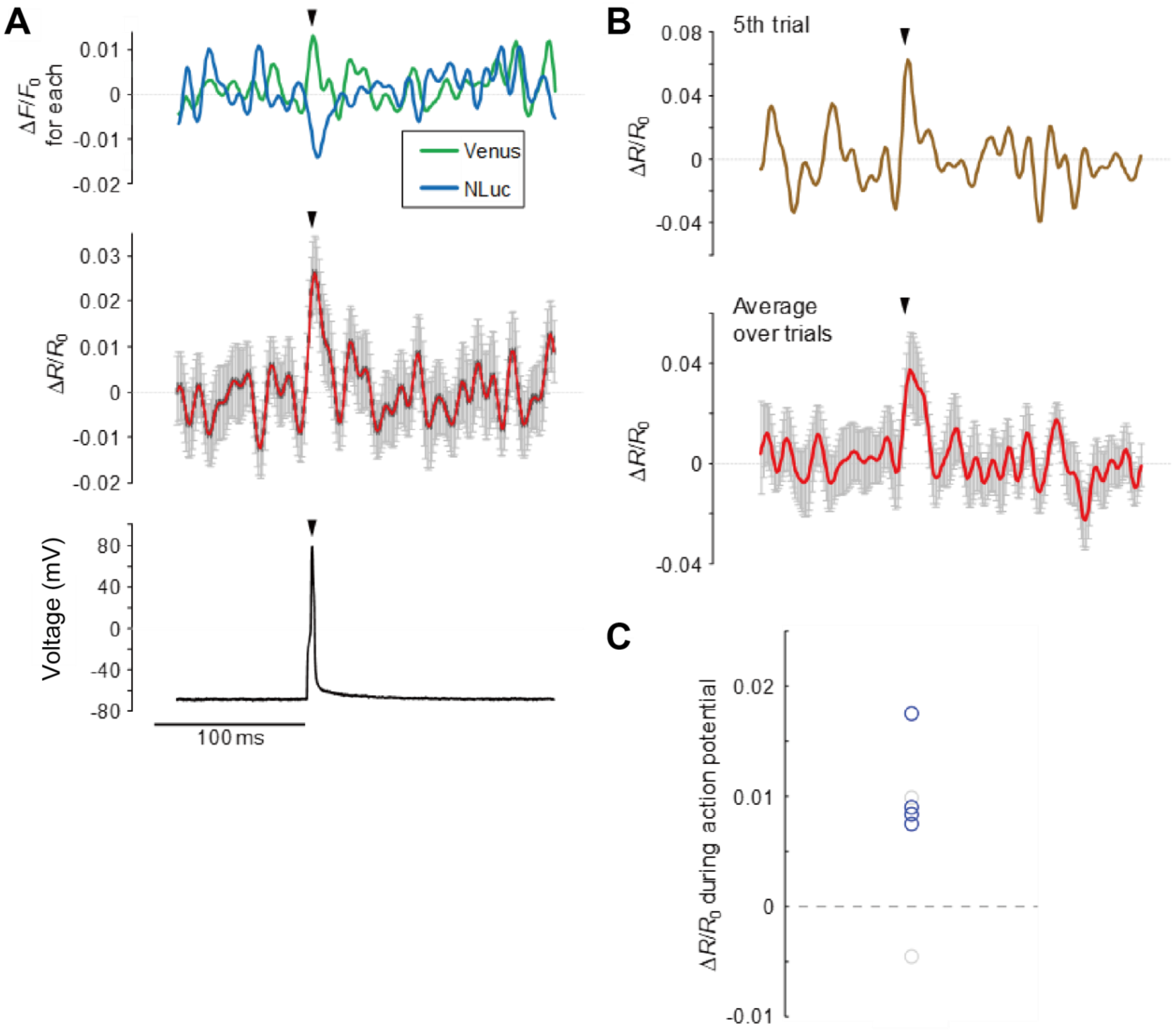

Supplementary Figure 2. Summary of LOTUS-V signal change in primary cultures of hippocampal neurons during neuronal firing. (**A**) Compared to the change in the respective Venus and NLuc signals (top), the change in $\Delta R/R_0$ (middle) during the action potential (bottom) was clearer. The average of recorded data over all trials (n=32 in this case) are shown in each panel. Arrow head, timing of the peak of the action potential. (**B**) In this example, even a single trial result (top) or small number of averaged trials (bottom, mean ± standard error over 8 trials) were sufficient to clearly demonstrated a signal change during the action potential. (**C**) Summary of the signal changes during the action potential (N=6 cells, average responses over trials). The majority of recorded cells independently demonstrated a significant increase of LOTUS-V signals during the action potential. Four of six samples demonstrated a statistically significant increase ($p<0.05$ Wilcoxon signed rank test), while one case showed a non-significant increase (p=0.059) and one showed a slight, but non-significant decrease (p=0.54))

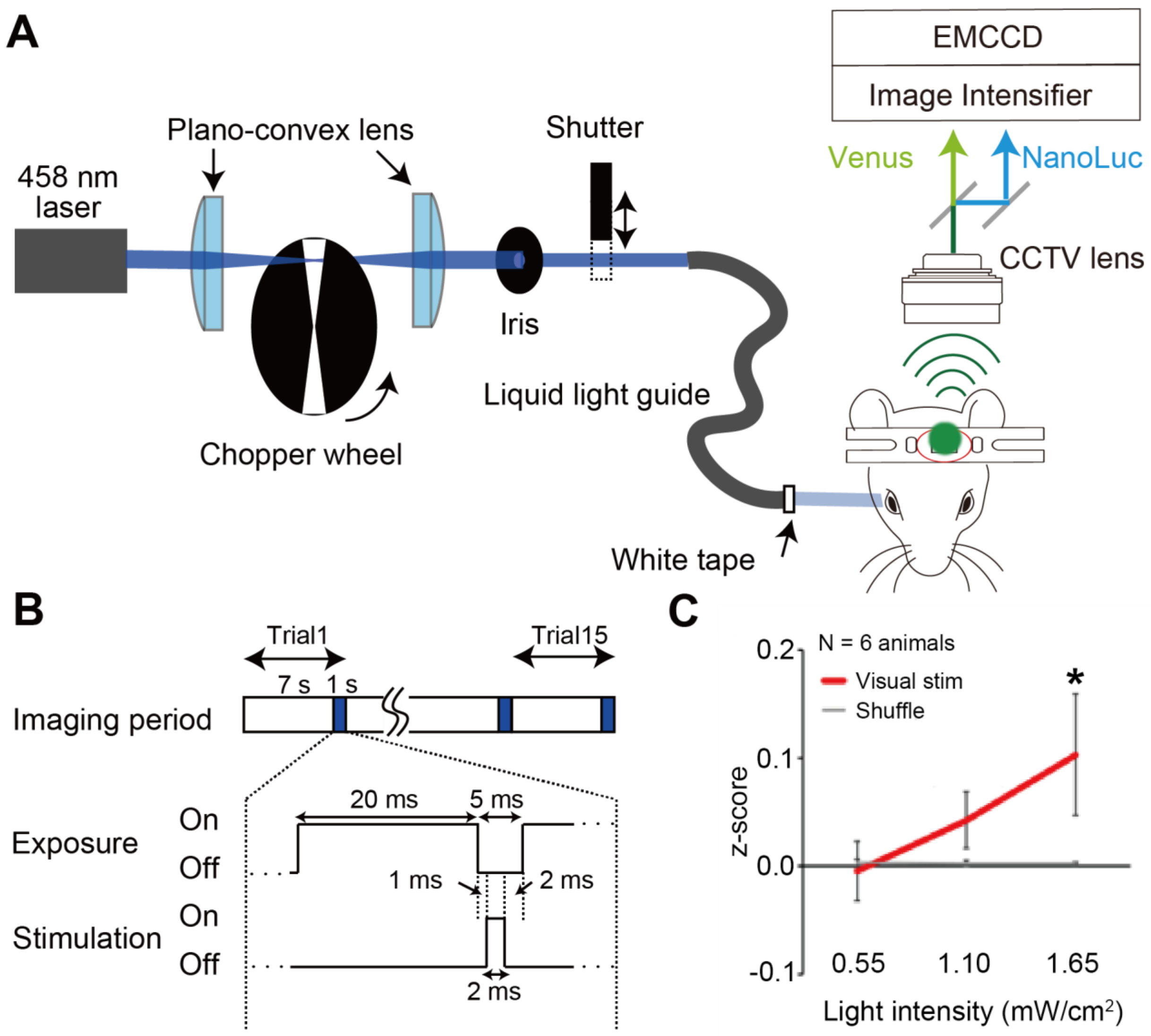

Supplementary Figure 3. LOTUS-V signal in the mouse primary visual cortex (V1) reported a significant increase during visual stimulation. (**A, B**) Optical design (**A**) and experimental configuration (**B**) of dead-time imaging of LOTUS-V bioluminescence signal (derived from the V1 of a head-fixed mouse) during a visual simulation paradigm. (**A**) The blue laser was coupled to a liquid light guide through two plano-convex lenses, a chopper wheel, iris diaphragm, and optical shutter to generate the light pulse. Bioluminescence from the V1 area was split by a dichroic mirror, and was separately recorded at the NLuc and Venus channels (<509 nm and ≥509 nm, respectively). (**B**) Exposure to the camera and visual stimulation during the processes for image readout and accumulated charge clearing on the camera (dead-time) were performed alternatively (blue; see also **Supplementary methods**). (**C**) There was a significant increase in LOTUS-V signals depending on the light intensities. Statistical analysis was conducted using a Wilcoxon signed rank test (N=6 mice, visual stimulation vs baseline at each light intensity). Although there were no significant differences from baseline with a weak light (0.55 mW/cm^2), the response was greater with increasing light intensity. Strong light (1.65 mW/cm^2) induced a significant change of the LOTUS-V signal. *; $p < 0.05$, n.s., not significant.

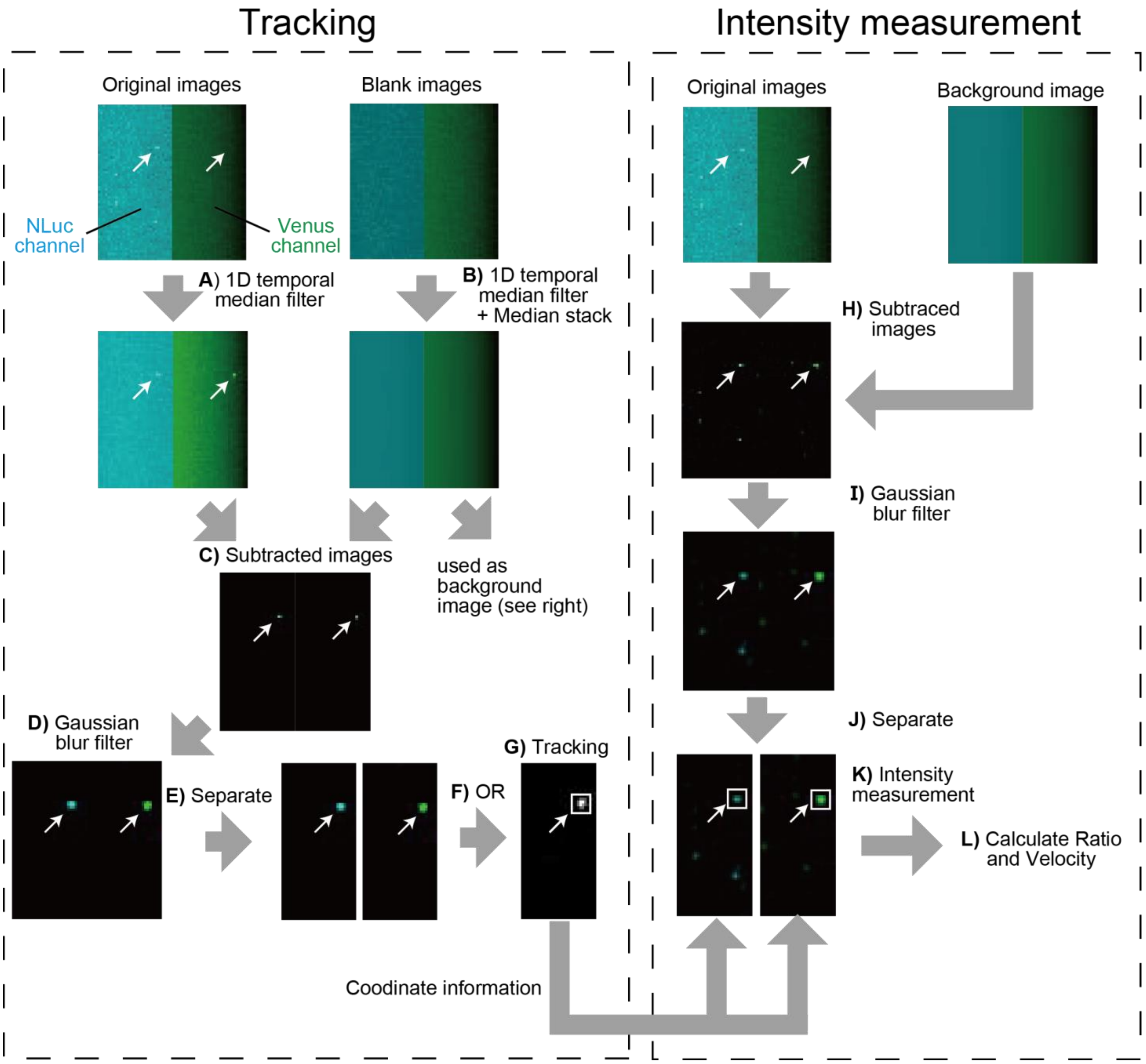

Supplementary Figure 4. Auto tracking method to obtain coordinate information and measure bioluminescence intensity of a freely moving mouse. The images contain two pieces of color information (green pixels, pseudo-colored Venus channel; cyan pixels, pseudo-colored NLuc channel) obtained using the image splitting optics, and thus bioluminescence from V1 of a freely moving mouse can be observed as two bright spots (indicated by white arrows) in each original image frame. Since we used a relatively high gain of the image intensifier (4-5 out of 10 levels) to decrease the exposure time and increase the frame rate, the effect of dark noise observed as bright spots apart from the brain signal was not negligible. Therefore, we separately performed “Tracking” and “Intensity measurement” steps to obtain precise coordinate information and bioluminescence intensity of the bright spots (see **Supplementary methods**). (**A**) To remove the dark noise using temporal information (the dark noise rarely appears at the same place in two consecutive frames), the 1D median filter was applied to the temporal axis. (**B**) To correct inhomogeneous background level, more than 1,000 blank images were acquired when the camera’s shutter was closed to make a “background image”. The 1D temporal median filter was also used to remove the dark noise. Finally, the median stack along the temporal direction was used as a background image. (**C**) This background image was subtracted from each median-filtered bioluminescent image to adjust the background level. (**D**) Gaussian blur filter was used in each image frame with a radius value set to 1.0, in order to easily

identify the signal using the Auto tracking program. (**E**) The images were separated into NLuc and Venus channels, and their x-y coordinates were aligned with each other by referring to the bright-field images we took before the experiments. (**F**) The separated channels were merged into single reference images containing intensity information of both channels using the "OR" function. (**G**) The coordinate information of the bright spot was obtained using the "Particle Track Analysis (PTA)" (Image J plug-in). This information was only used to set the ROI on the bright spot and measure the signal intensity of Venus and NLuc channels at the following "intensity measurement" steps. (**H**) The original images "without" the 1D temporal median filter were background-subtracted. (**I**) Gaussian blur filter was used. (**J**) The images were separated into NLuc and Venus channels. (**K**) The intensities of the bright spot in both channels were measured by locating the ROI based on the coordinate information obtained by PTA (**G**). (**L**) The ratio and velocity were calculated after measuring the bioluminescence intensity of both channels.

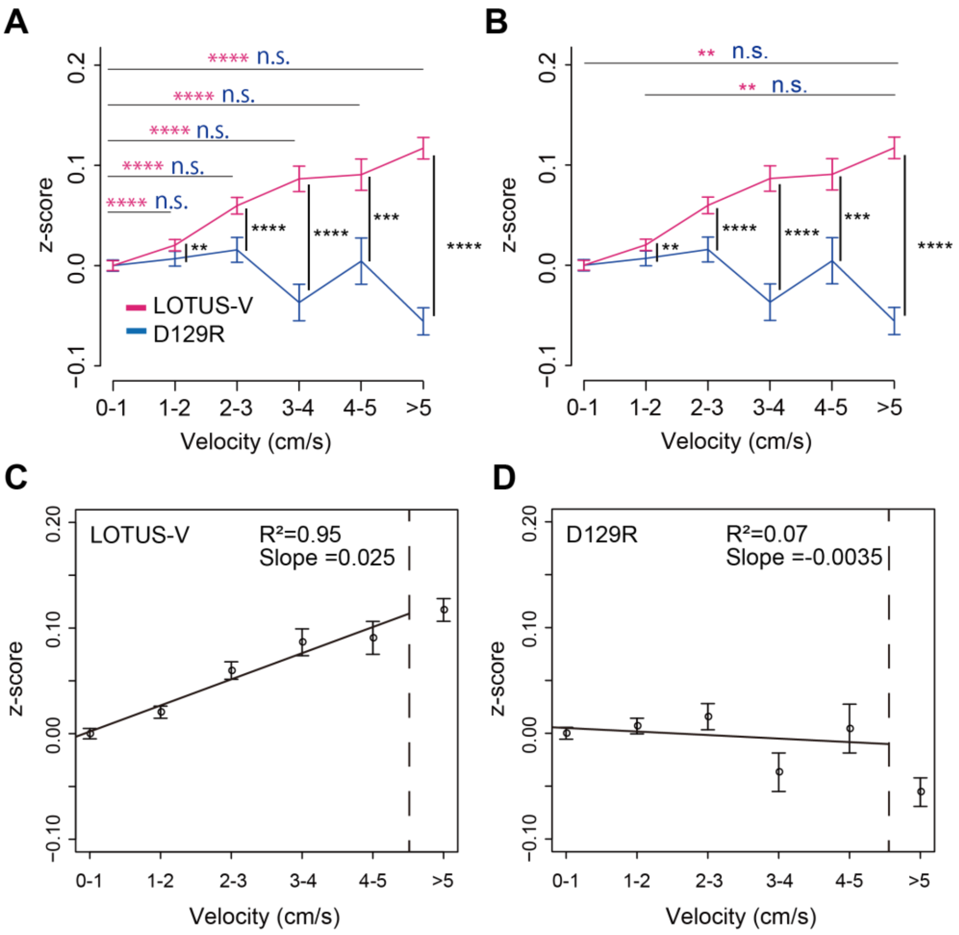

Supplementary Figure 5. Evaluation of the locomotion-dependent artifact in LOTUS-V signal. (A, B) To consider locomotion driven artifact, we compared signals of LOTUS-V with those of voltage-insensitive mutant, LOTUS-V(D129R) after categorizing data by locomotion speed of the mouse. The plots show z-normalized $\Delta R/R_0$ from LOTUS-V (magenta) or LOTUS-V(D129R) (blue) -expressing and single-housed freely-moving mice in each velocity category (LOTUS-V, n =41005, 29712, 14255, 7296, 4889 and 13674 time-points from N =5 mice, for 0-1, 1-2, 2-3, 3-4, 4-5 and >5 cm/s, respectively; D129R, n =31648, 17771, 6418, 2889, 1949 and 4979 time-points from N=3 mice). (**A**) p <0.0001 and <0.01 for LOTUS-V and D129R respectively for Kruskal-Wallis test with all six categories, whereas the results of multiple comparison (calculated by Steel-Dwass test) shown in this panel as blue or magenta indicates substantial increase in LOTUS-V and no significant difference in LOTUS-V(D129R). We also performed Wilcoxon rank sum test to indicate the statistical differences between LOTUS-V and LOTUS-V(D129R), suggesting the locomotion-drive signal change in LOTUS-V was not the simple locomotion artifact. (**B**) In addition, we applied a linear mixed-effect model to consider the possibility that the samples from each mouse were correlated. ANOVA for the linear mixed-effect model indicates p<0.05 and n.s. for LOTUS-V and D129R, respectively. The results of the Tukey multiple comparison test for a linear mixed-effect model are shown in this panel as blue or magenta. (**C, D**) The plots from LOTUS-V (**C**) and LOTUS-V(D129R)-expressing mice (**D**) were line fitted by the least-squares method (without using the values in the over 5 cm/s category). Time bin, 0.1 s; n.s., not significant; **, p <0.01; ***, p <0.001; ****, p <0.0001.

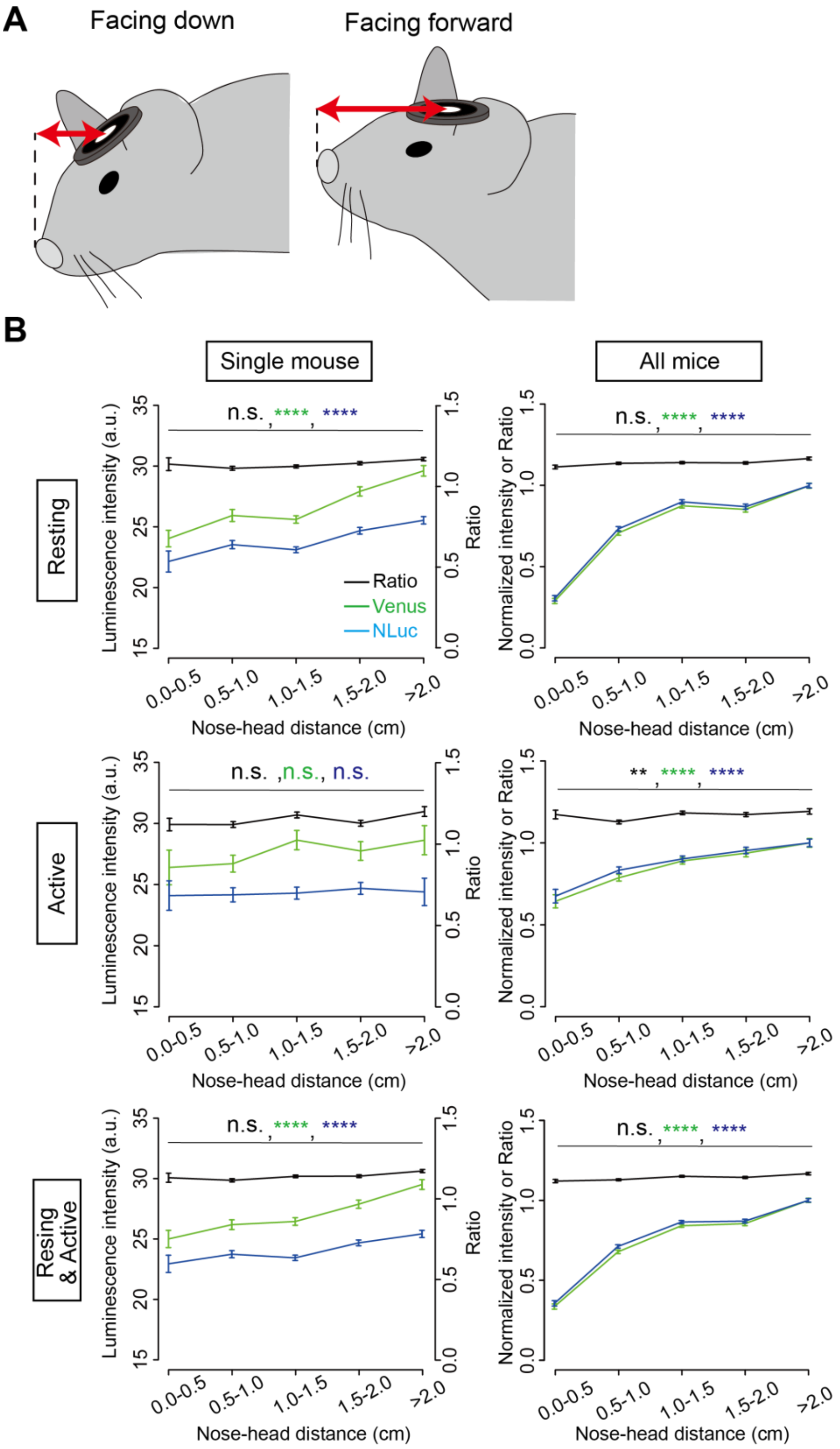

Supplementary Figure 6. The head-angle dependent artifact was removed by calculating ratio. (**A**) The schematic illustrations show how we measured the length from the nose to the cranial window (nose-head length, red arrows). Since this length of each mouse during free movement changes depending on the head angle, we calculated this value and compared it with signal from the brain. (**B**) Plots indicate NLuc (blue), Venus (green) and ratio (black) signals in each nose-head length category, in (left) a single representative mouse (resting, n =52, 378, 748, 782 and 858 time-points for 0.0-0.5, 0.5-1.0, 1.0-1.5, 1.5-2.0 and >2 cm, respectively; active, n =36, 182, 289, 214 and 98 time-points; resting and active,

n =88, 560, 1037, 996 and 956 time-points), or (**right**) all mice (resting, n =473, 1287, 1490, 1295 and 1067 time-points from N =3 mice; active, n =116, 549, 799, 514 and 342 time-points; resting and active, n =589, 1836, 2289, 1809 and 1409 time-points). In a right panel**,** Venus and NLuc signals were normalized by the averages of the longest category (i.e. when the mouse head was directed horizontally) respectively. Ratio was calculated by dividing pre-normalized Venus signal by pre-normalized NLuc signal. Data not during the interaction with other mice (i.e. when distance between them was more than 4cm) was used. P-values were obtained using Kruskal-Wallis test with all five categories. In addition, we applied ANOVA for a linear mixed-effect model to consider the possibility that samples from each mouse were correlated. We found no significant difference between ratio values in each category (Resting, p=0.553; Active, p=0.053; Resting & Active, p=0.160). Time bin for the analyses, 0.1 s; Error bars indicate mean ± standard error; n.s., not significant; **, p<0.01; ****, p <0.0001.

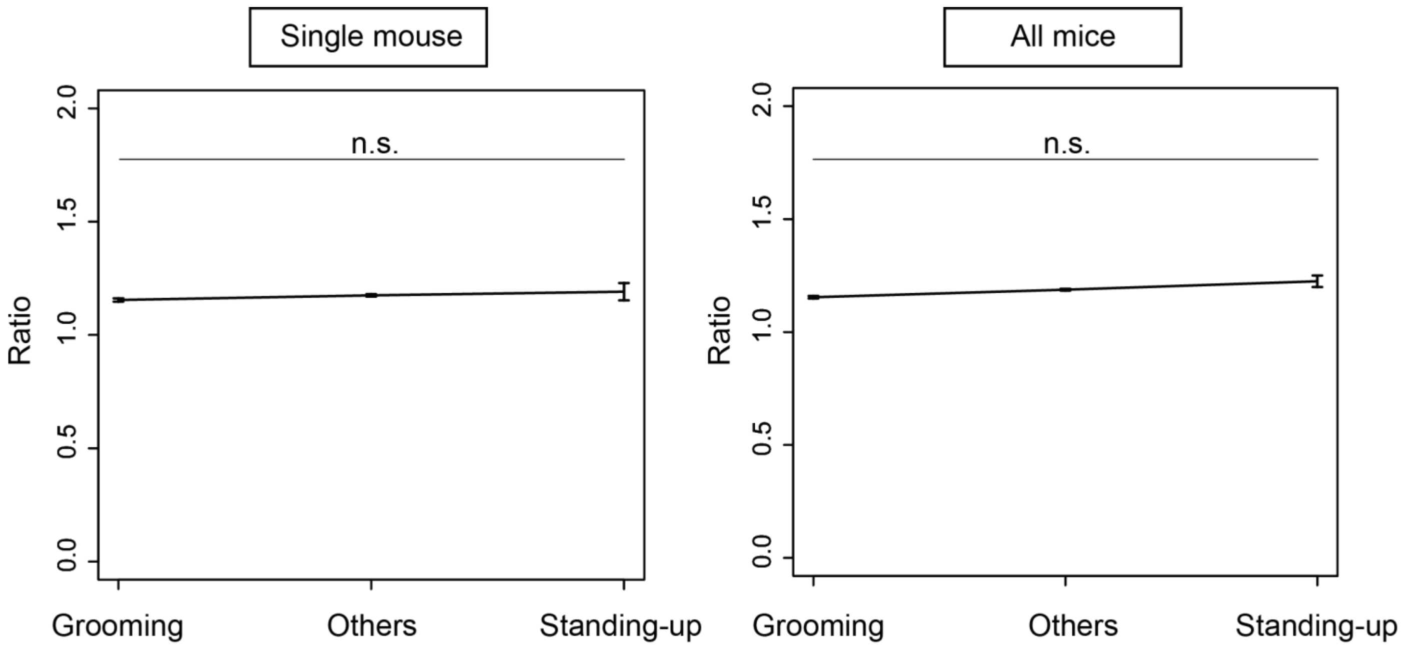

Supplementary Figure 7. Vertical motion does not affect the LOTUS-V signal. To confirm whether vertical motion affected the LOTUS-V signal, the ratio value of the LOTUS-V signal during mouse grooming and standing-up was evaluated in a single representative mouse (left; n =1334, 2880 and 64 time-points for grooming, others and standing-up, respectively), or all mice (right; n =2407, 5396 and 164 time-points, respectively). Data during the interaction with other mice (i.e., when distance between them was more than 4 cm) were not used. P-values were obtained using the Kruskal–Wallis test (**left**) and ANOVA for a linear mixed effect model (**right**). Time bin for the analyses, 0.1 s; Error bars indicate mean ± standard error; n.s., not significant.

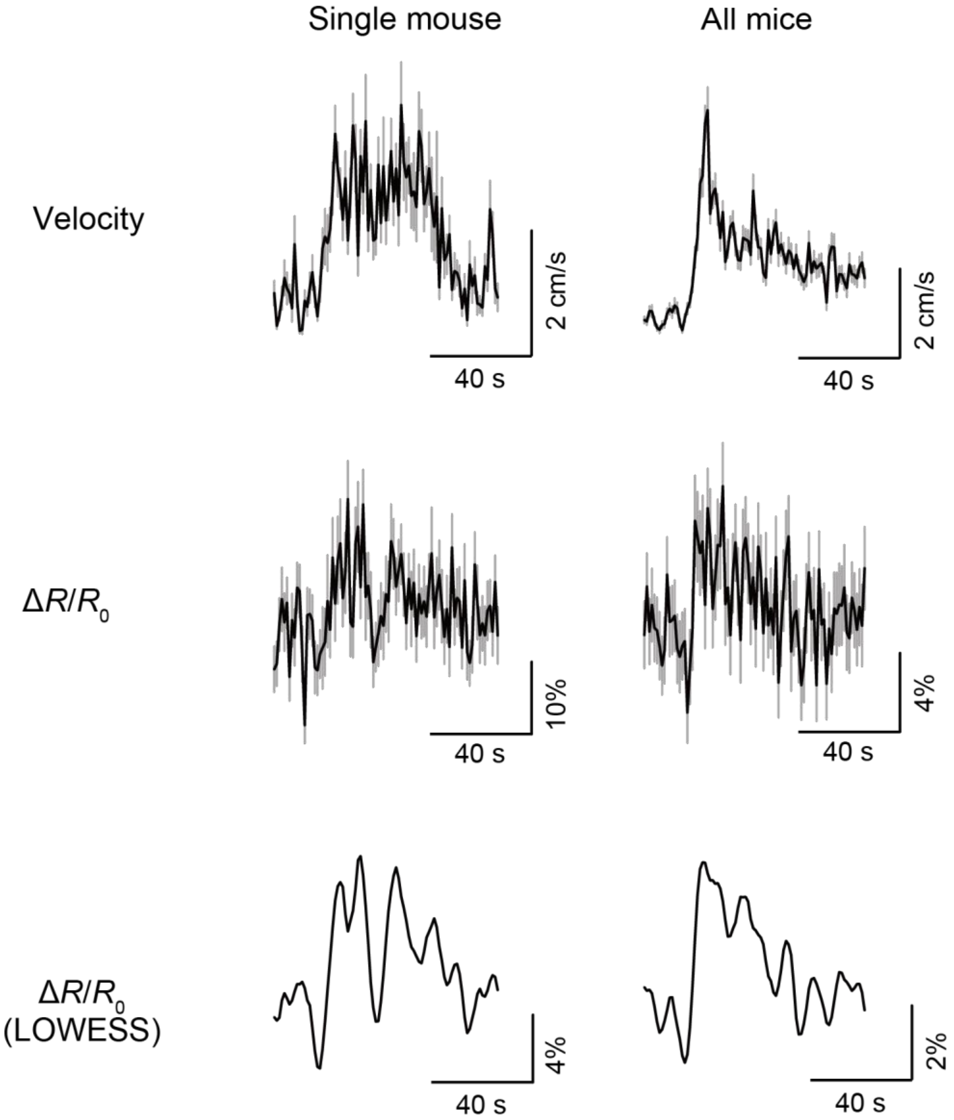

Supplementary Figure 8. Manual tracking data also showed dynamic change in LOTUS-V signal in V1 after the locomotion onset. Averaged time series of locomotion velocity (upper panels) and LOTUS-V signal $\Delta R/R_0$ (middle) of a single representative mouse (left, n =6 sessions) and all mice (right, n =27 sessions from N =3 mice) around the locomotion onset were plotted. Manual tracking data were exclusively used for this analysis. Lower panels show LOWESS-smoothed $\Delta R/R_0$. The Granger causality test statistically revealed the strong link between the locomotion velocity and the LOTUS-V signal ($p<0.0001$ for both “representative mouse” and “all mice”), suggesting that the change in the velocity might trigger the change in $\Delta R/R_0$. Time bin, 1 s; Error bars indicate mean ± standard error.

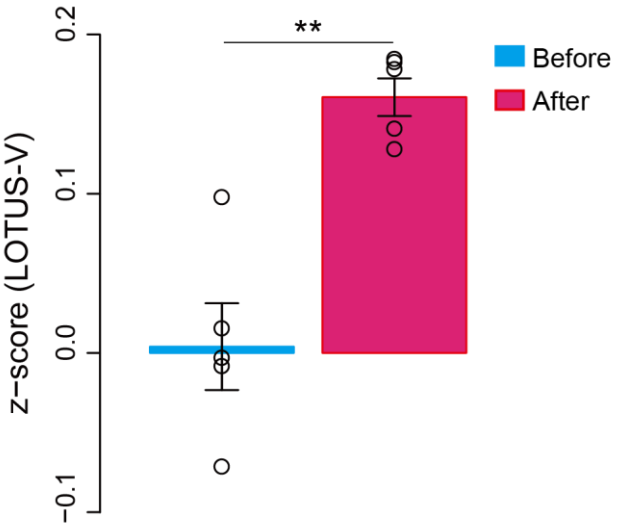

Supplementary Figure 9. Statistical analysis of the signal change before and after the locomotion onset using manual tracking data. The averages of z-normalized $\Delta R/R_0$ of LOTUS-V expressing mice before (-19 s to 0 s) and after (1 s to 20 s) the locomotion onset were shown as bar graphs, while each circle indicates the value at each time point of "All mice" (shown in **Supplementary Fig. 7)**. p-value shown in the panel was calculated using a Wilcoxon rank sum test. Time bin, 4 s; Error bars indicate mean ± standard error; n.s., **, p<0.01.

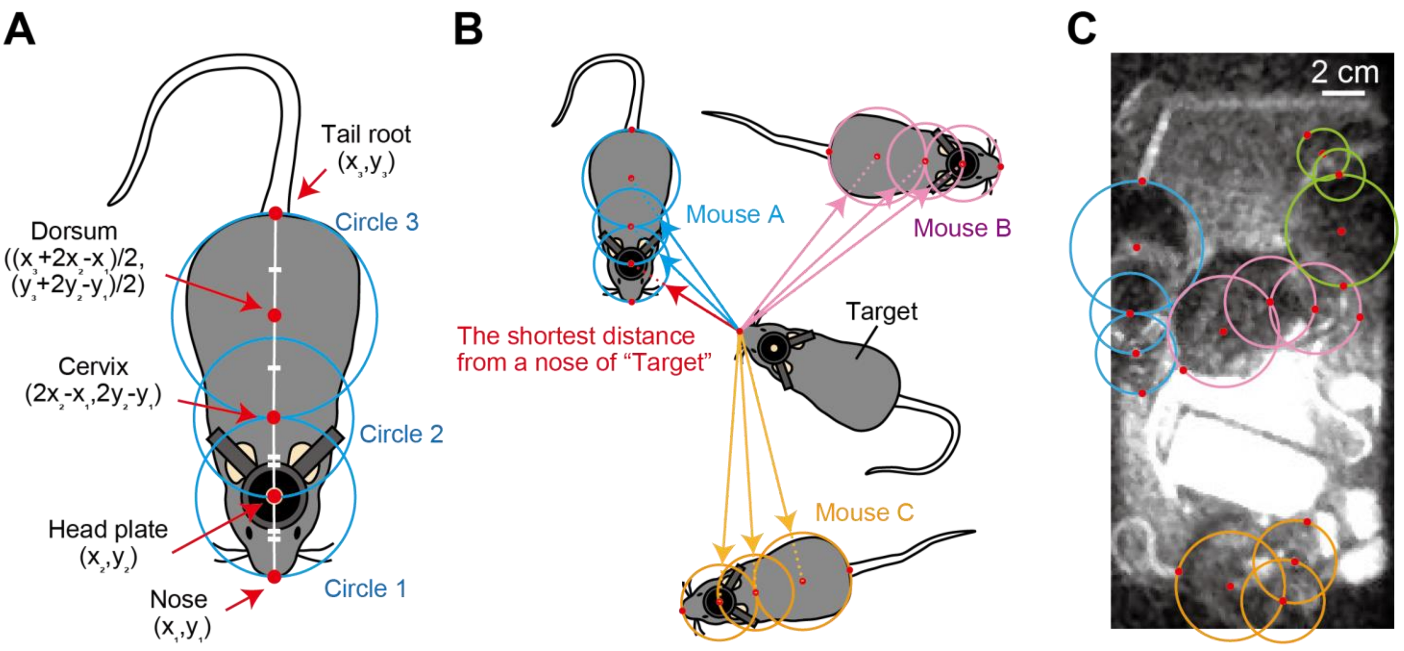

Supplementary Figure 10. Measurement of the distance between interactively locomoting mice. (**A**) Image illustrating the definition of the approximate areas of the mouse. The nose, center of the headplate, and tail root positions were manually tracked. This coordinate information was used to calculate the positions of the cervix and dorsum as indicated. The centers of the three variable circles were located at the headplate (Circle 1), cervix (Circle 2), and dorsum (Circle 3), respectively, as shown in the panel. The radius of each circle was set as the distance between the headplate and nose (i.e., Circle 1 and Circle 2, respectively), and the dorsum and tail root (Circle 3). (**B**) An image illustrating how the distances between the target mouse and other mice, interacting with one another, at each time-point were calculated. The distances between the nose of the target mouse and the closest edge of each circle were also calculated. Among them, the shortest distance was used as the score for the interaction analysis (red arrow). When the nose was the inside of the circle, the distance was represented as a negative value. (**C**) An example of an overlaid image of the bright field and approximate mouse areas. The color of each circle indicates the different individual mice.

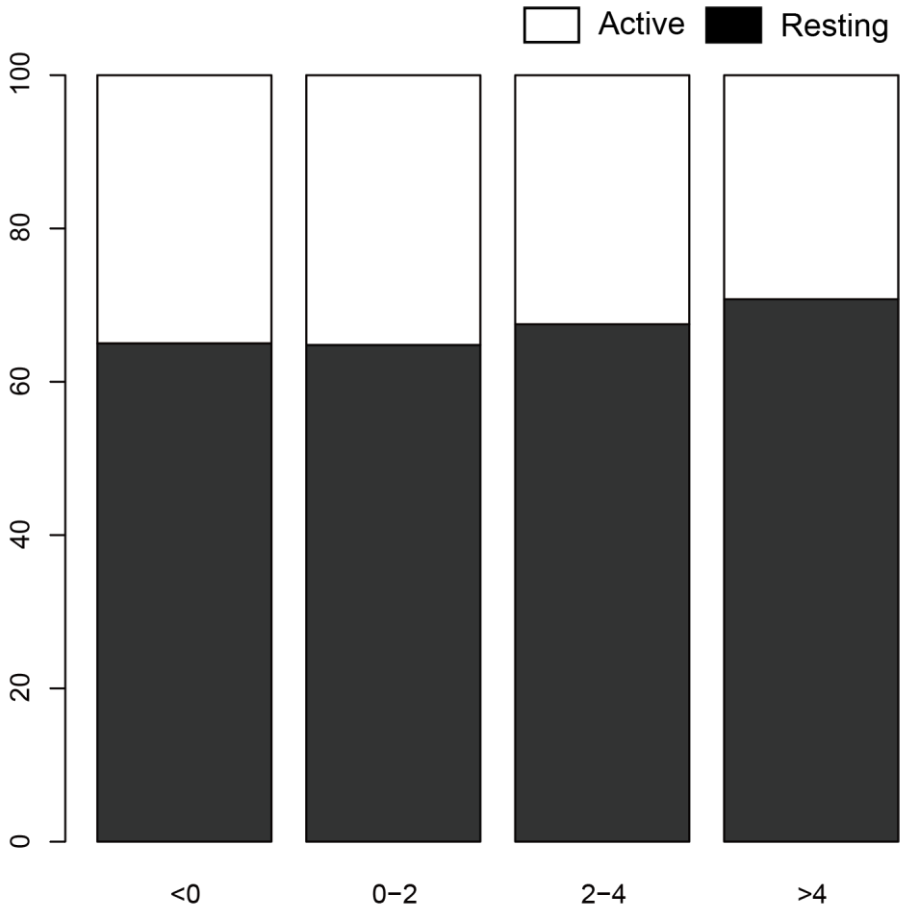

Supplementary Figure 11. The fraction of resting and active states in each distance category. Data from all mice are summarized (the resting state, n =3538, 7606, 5843, and 5612 time-points for <0, 0–2, 2–4, and >4 cm, respectively; the active state, n =1905, 4133, 2814, and 2320 time-points, respectively). The fractions of resting and active states were indicated as black and white boxes in the 100% stacked bar chart, respectively. Each bar corresponds to the distance category between the target mouse and closest mouse (see also **Supplementary Fig. 10**).

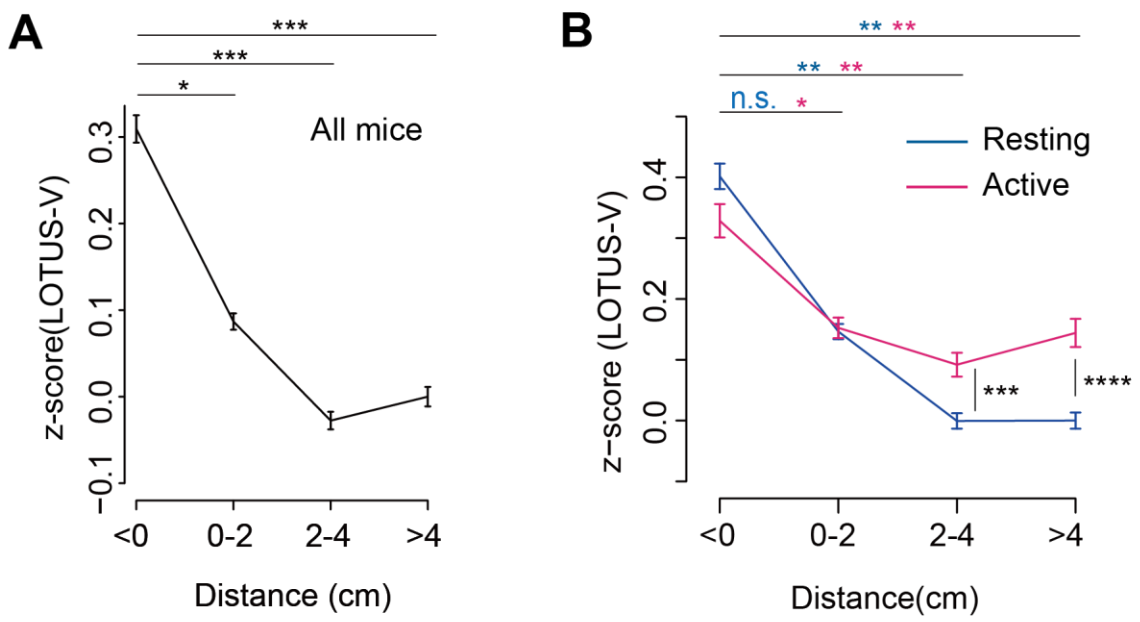

Supplementary Figure 12. Analysis of interactively moving mice with a linear mixed-effect model. To consider that the samples from each mouse are correlated, we analyzed the data in **Fig. 4C and D** with a linear mixed-effect model. (**A**) Distance-dependent change in the activity of the primary visual cortex (V1) of interactively locomoting mice. Plots represent z-normalized $\Delta R/R_0$ of each distance category (distance between the target mouse nose and other mice, see also **Fig.4C** and **Supplementary Fig. 10**; n= 5443, 11739, 8657, and 7932 time-points for <0, 0–2, 2–4 and >4 cm, respectively). The distant values (>4 cm) were used as a baseline (R_0) to calculate the z-normalization. p<0.05 for ANOVA for a linear mixed-effect model. P-values shown in the panel were calculated using the Tukey multiple comparison test for a linear mixed-effect model. (**B**) Comparison of distance-dependent change in V1 activity and the effect of locomotion (resting vs active states) (see also **Fig.4D)**. Data from all mice were used (resting state (<1 cm/s, blue), n= 3538, 7606, 5843, and 5612 time-points for <0, 0–2, 2–4, and >4 cm, respectively; active state (>1 cm/s, magenta), n= 1905, 4133, 2814, and 2320). The $\Delta R/R_0$ in the “distant and resting” state was used as the baseline for z-normalization. P-values obtained using ANOVA for a linear mixed-effect model were used; p <0.05 in both states. P-values obtained using the Tukey multiple comparison test for a linear mixed-effect model are shown as blue (resting states) or magenta (active) symbols, while those obtained using the Wilcoxon rank sum test (to compare resting vs active) are shown in black. The time bin was 0.1 s. Error bars indicate mean ± standard error; n.s., not significant; *, p <0.05; **, p <0.01;***, p <0.001; ****, p <0.0001.

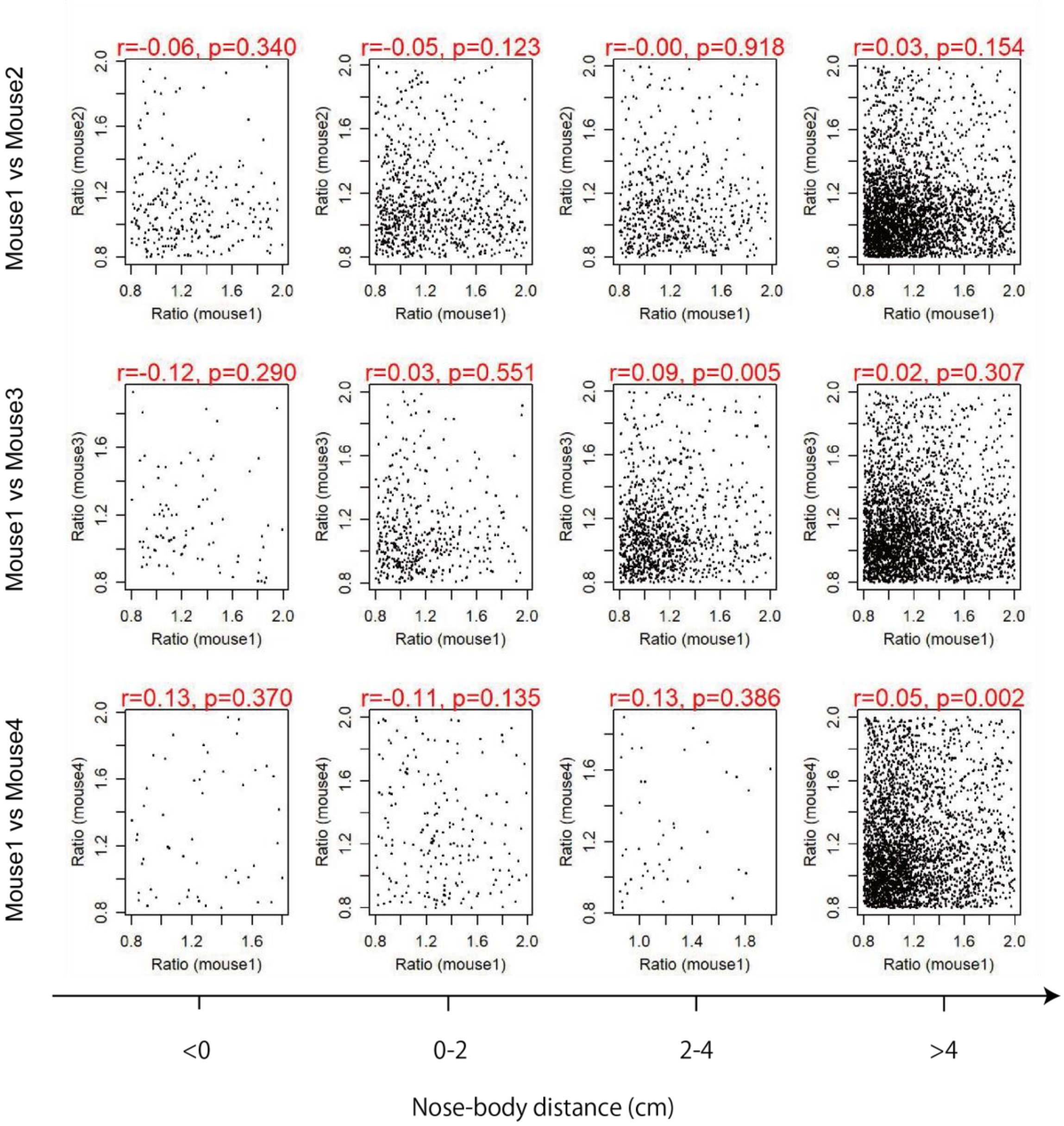

Supplementary Figure 13. Test to confirm whether signal from one mouse might be leaked to the others of the close distance (Mouse1's nose to others' bodies). The scatter plot indicates the relationship of brain signals (Venus/NLuc ratio) from two different mice in the same cage, at each distance category (Mouse1 vs Mouse2, n =233, 691, 466, and 2553 time-points for <0, 0–2, 2–4 and >4 cm, respectively; Mouse1 vs Mosue3, n =75, 365, 868, and 2477; Mouse1 vs Mouse4, n =43, 155, 41 and 2531). The distances were calculated as described in **Supplementary Fig. 10**. Pearson correlation coefficient (r) and p-value (p) from Pearson correlation test are indicated in each panel.

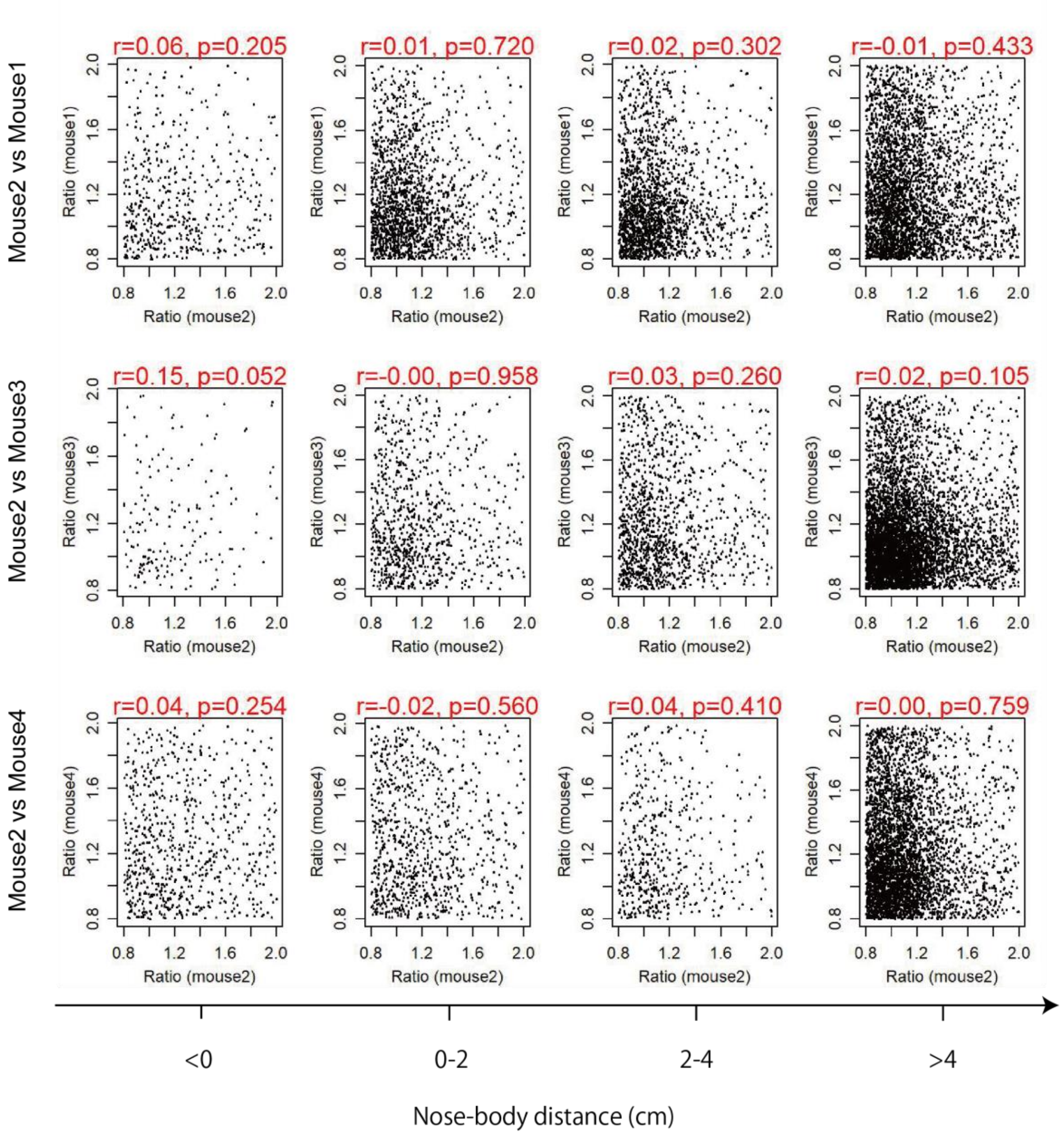

Supplementary Figure 14. Test to confirm whether signal from one mouse might be leaked to the others of the close distance (Mouse2's nose to others' bodies). The scatter plot indicates the relationship of brain signals (Venus/NLuc ratio) from two different mice in the same cage, at each distance category (Mouse2 vs Mouse1, n =391, 1371, 1521, and 2923 time-points for <0, 0–2, 2–4 and >4 cm, respectively; Mouse2 vs Mosue3, n =134, 625, 951, and 4392; Mouse2 vs Mouse4, n =539, 682, 399 and 3242). The distances were calculated as described in **Supplementary Fig. 10**. Pearson correlation coefficient (r) and p-value (p) from Pearson correlation test are indicated in each panel.

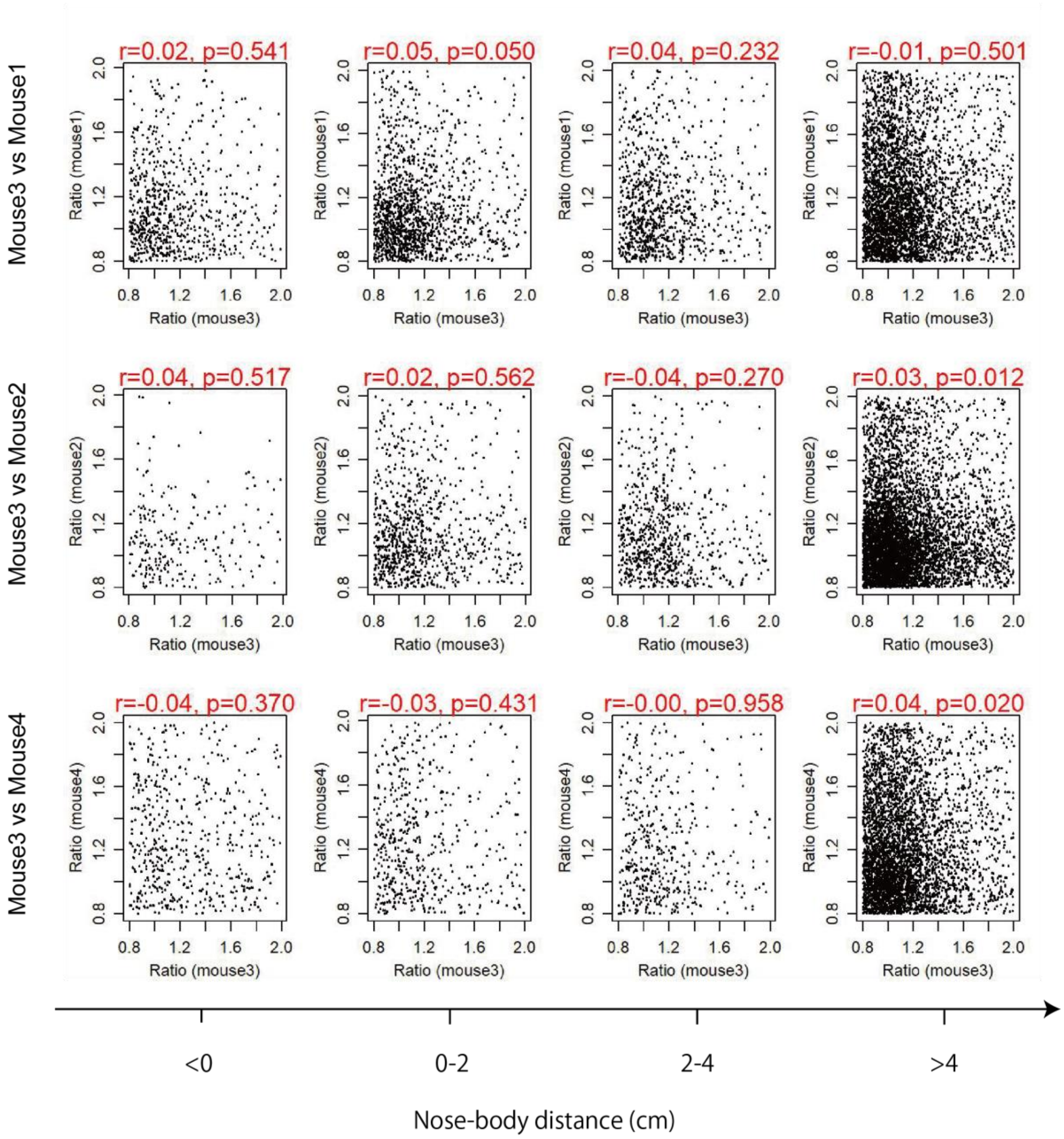

Supplementary Figure 15. Test to confirm whether signal from one mouse might be leaked to the others of the close distance (Mouse3's nose to others' bodies). The scatter plot indicates the relationship of brain signals (Venus/NLuc ratio) from two different mice in the same cage, at each distance category (Mouse3 vs Mouse1, n =584, 1154, 768, and 3035 time-points for <0, 0–2, 2–4 and >4 cm, respectively; Mouse3 vs Mosue2, n =188, 664, 597, and 4261; Mouse3 vs Mouse4, n =400, 383, 349 and 3237). The distances were calculated as described in **Supplementary Fig. 10**. Pearson correlation coefficient (r) and p-value (p) from Pearson correlation test are indicated in each panel.

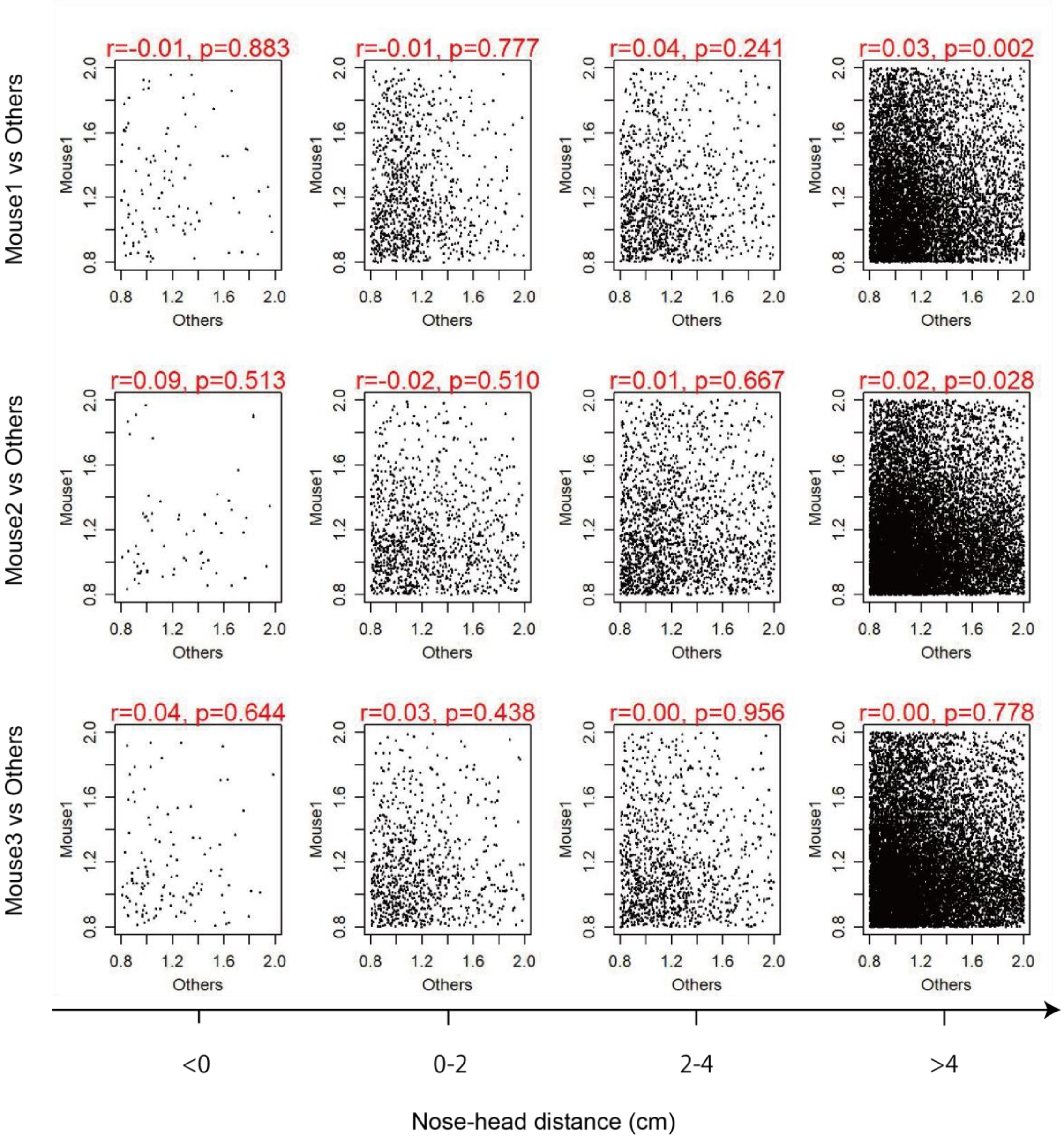

Supplementary Figure 16. Test to confirm whether the signal from one mouse might be leaked to others because of the close distance (Mouse nose to others' head). When the heads of two mice were closed, the signal could be more easily contaminated into another one. To test this possibility, the distances between the nose of the target mouse and the edge of the circle located on the head of the other mouse were calculated as described in **Supplementary Fig. 10**. The scatter plot indicates the relationship of brain signals (Venus/NLuc ratio) from two different mice in the same cage, according to each distance category (Mouse 1's nose vs other's head, n =101, 940, 1096, and 8361 time-points for <0, 0–2, 2–4 and >4 cm, respectively; Mouse 2's nose vs other's head, n =60, 1070, 1798, and 14242; Mouse3's nose vs other's head, n =115, 852, 1146 and 13507). Pearson's correlation coefficient (r) and p-value (p) from the Pearson's correlation test are indicated in each panel.

Supplementary Video 1. Imaging of a single mouse expressing LOTUS-V in the primary visual cortex (V1). Overlay of bright field and LOTUS-V bioluminescence (green).

Supplementary Video 2. Trajectory movie of the locomotion velocity and z-normalized $\Delta R/R_0$ in a single mouse recording. Pseudo-color (blue to red) indicates 0 to 20 cm/s for velocity and -2.5 to 2.5 for z-normalized $\Delta R/R_0$, respectively.

Supplementary Video 3. Imaging of multiple mice expressing LOTUS-V in the primary visual cortex (V1). Overlay of bright field and LOTUS-V bioluminescence (green).

Supplementary Video 4. Trajectory movie of locomotion velocity and z-normalized $\Delta R/R_0$ in multiple mice recording. Pseudo-color (blue to red) indicates 0 to 20 cm/s for velocity and -1 to 1 for z-normalized $\Delta R/R_0$, respectively.

Supplementary methods

AAV preparation. For the AAV expression system, the cDNA of LOTUS-V (GenBank accession number; LC061443) and LOTUS-V(D129R) were amplified from pcDNA3-CMV-LOTUS-V and pcDNA3-CMV-LOTUS-V(D129R) respectively, by polymerase chain reaction, using a sense primer, containing a Kozak sequence following a *Bgl*II site, and a reverse primer, containing a *Hin*dIII site and a stop codon. They were then ligated with pAAV2-hSyn [2] digested by *Bam*HI and *Hin*dIII. DNA sequencing was used to verify all constructs.

The AAV-DJ vector was prepared as described previously with some modifications [3]. Briefly, HEK293T cells (RIKEN BRC Cell Bank, RCB2202) were grown in Dulbecco's modified Eagle's medium (Sigma) containing heat-inactivated 10% FBS (Biowest) at 37 °C in 5% CO_2. Equal amounts of pAAV2, pAAV-DJ [4], and pHelper (Cell Biolabs, INC.) plasmids were co-transfected using FuGENE$_{HD}$ transfection reagent (Promega), following the manufacturer's protocol. The cells were trypsinized and centrifuged for 5 min at 1000 rpm at 4 ºC, 3 days after infection. The pellet was resuspended in 200 µl HEPES-buffered saline (10 mM HEPES, pH 7.3, containing 150 mM NaCl, 2.5 mM KCl, 1 M $MgCl_2$, and 1 M $CaCl_2$) and subjected to three freeze-and-thaw cycles. Then, 1 µl of benzonase nuclease was added to each tube, warmed in a water bath at 45 ºC for 15 min, and centrifuged for 10 min at 16,000 *g* at 4 ºC. The supernatant was transferred into a new Eppendorf tube and centrifuged for 10 min at 16,000 *g* at 4 ºC. Thereafter, the supernatant was transferred to a new Eppendorf tube and aliquots were stored at -80 ºC until use.

Rat hippocampal neuron culture for imaging. Primary cultures of hippocampal neurons and astrocytes were prepared from embryonic day 17 Sprague-Dawley rats. Cells were dissociated in plating medium consisting of Hanks' Balanced Salt

solution (HBSS; Wako), which was supplemented with 1 mM HEPES and 100 U/ml penicillin/streptomycin. The cells were then plated on a poly-L-lysine (Sigma) coated 35-mm dish with a coverslip bottom at a density of 3.5×10^4 cells/12-mm-diameter coverslip. The medium was changed to culture medium constituting of Neuro Basal (Thermo Fisher), supplemented with 2% B27 (Invitrogen) and L-glutamine, 5 h after plating, and the cultures were grown in 5% CO_2 at 37 °C. On day 7 *in vitro* (DIV-7), half of the medium was replaced with fresh culture medium. On DIV-14, the stock solution containing AAV vector was mixed with culture medium up to 1.0×10^{10} TU/ml. After 5 h of incubation, the medium was replaced to fresh culture medium. Cultures were incubated again at 37 °C in 5% CO_2, and were used for experiments 7–10 days after infection.

Electrophysiology and photometry. LOTUS-V-expressing hippocampal cultured neurons were subjected to simultaneous patch-clamp and FRET recordings at 7–10 days post-infection. The medium was replaced with a bath solution consisting of HBSS (Gibco) supplemented with 20 mM HEPES (pH 7.2) and 5.5 mM D-Glucose, and the dish was mounted on an Axiovert 200M inverted microscope (Carl Zeiss). Patch-clamp recordings in the whole-cell mode were made using an Axoclamp 200B patch-clamp amplifier, with a capacitive head stage (Axon Instruments), using glass recording electrodes (3–5 MΩ) filled with intracellular solution (i.e., 140 mM potassium gluconate, 5 mM KCl, 1 mM $MgCl_2$, 0.1 mM EGTA, 2 mM Mg-ATP, 5 mM HEPES, adjusted to pH 7.2 with KOH). Whole-cell recordings were low-pass-filtered at 1 kHz and digitized at 10 kHz. Data were digitized with a Digi data 1342 digitizer (Axon Instruments) and fed into a computer for offline analysis using AxoClamp 9.0 software (Axon Instruments). Bioluminescence of LOTUS-V was observed by adding 50 μM furimazine (Promega) and recordings were performed at 23°C. Images of the Venus and NLuc channels were acquired simultaneously using W-VIEW GEMINI image splitting optics (Hamamatsu Photonics), C8600-05 GaAsP image intensifier unit (Hamamatsu Photonics), and MiCAM Ultima-L CMOS camera (Brain Vision). The image splitting optics contained a FF509-FDi01-25×36 dichroic mirror (Semrock) and no emission filters. The optical signal was analyzed offline using BrainVision analyzer software.

Preparation for *in vivo* voltage imaging. Viruses were injected to C57BL/6JJmsSlc mice at postnatal day P35-40 for *in vivo* experiments. Procedures were conducted as previously described [5], with some modification. During surgery, mice were anesthetized with isoflurane (initially 2% partial pressure in air, and reduced to 1%). A small circle or a square (~1 mm in diameter) was thinned over the left V1 using a dental drill to mark the site for craniotomy and target the putative monocular

region. AAV-DJ vector was injected into the left V1 (2.1 mm lateral to the midline, 0.3 mm rostral to lambda at a depth of 300 µm), over a 15-min period, using a UMP3 microsyringe pump (World Precision Instruments). The total volume of the AAV crude solution was 375 nl. The beveled side of the needle was faced to the left so that viruses could be injected into and cover the V1 area of the left hemisphere.

Mice were anesthetized by isoflurane (1–1.5%), at 3 weeks to 5 months after the virus injection, before the *in vivo* recording. *In vivo* two-photon imaging was performed as previously described [5] to confirm the expression level of LOTUS-V. Briefly, a titanium headplate was attached to the skull using dental cement, and the cranial window was made over the V1, around the virus injection site (1.5 mm in diameter) for subsequent imaging. The brain surface was covered with 4% agarose gel dissolved in HEPES-buffered saline (10 mM HEPES, pH 7.3, containing 150 mM NaCl, 2.5 mM KCl, 1 M $MgCl_2$, and 1 M $CaCl_2$). HEPES-buffered saline was poured over the gel until we initiated bioluminescence imaging. The level and areas of expression were confirmed by imaging Venus signals with a FVMPE-RS two-photon microscope (Olympus) and a Mai Tai DeepSee Ti:sapphire laser (Spectra-Physics) at 920 nm, through a 4x dry objective, 0.28 N.A. (Olympus) or a 25x water immersion objective, 1.05 N.A. (Olympus). Scanning and image acquisition were controlled using FV30S-SW image acquisition and processing software (Olympus).

For the bioluminescence imaging, a solvent of furimazine solution (Nano-Glo luciferase assay system, Promega) was evaporated with a VDR-20G vacuum desiccator (Jeio Tech) and a BSW-50N belt drive rotary vane vacuum pump (Sato Vac Inc.) overnight under dark conditions. The precipitate was eventually dissolved in propylene glycol (up to 5 mM). This solution was kept at -30 °C as a stock solution and used for further imaging experiments.

For *in vivo* imaging from awake mice, we previously injected the substrate for the bioluminescence into mice through intraperitoneal or intravenous injection[6]. However, in these cases, it was difficult to introduce the high concentration substrate into the V1 area. Therefore, here we used a substrate-enclosed cranial window to directly deliver the substrate (~50 µM) into the target brain area. We attached a small o-ring (diameter: 10 mm) on the headplate using Kwik-Sil silicon adhesive (World Precision Instruments), as described in **Fig. 2D**. In some experiments, we alternatively used headplates consisting of an o-shaped window with a certain thickness (and two side bars to hold the mouse head) [7]. This circular pool area was used to keep the furimazine solution over the cranial window (~200 µl). During the bioluminescence imaging, the HEPES-buffered saline over the agarose gel was replaced with 50 µM furimazine solution (dissolved in 200 µl of the HEPES-buffered saline). This o-ring pool was eventually covered by a cover glass (15 mm

diameter) glued over the o-ring with the Kwik-Sil adhesive. To minimize the reflection of the bioluminescence from LOTUS-V-expressing neurons at the outside of the brain, the surrounding areas, including surface of the headplate and dental cement, were stained black using Touch Up Paint X-1 matte black (SOFT99). This was important to suppress noisy signals from the target area. Further, the inside of the mouse chamber that was used for imaging during free movement was stained black using a black guard spray (Fine Chemical Japan).

***In vivo* imaging of head-fixed mice.** For signal detection, we used a Lumazone *in vivo* luminescence imaging system (Molecular Devices) equipped with Evolve Delta 512 EMCCD camera (Photometrics), C8600-05 GaAsP high-speed-gated image intensifier unit (Hamamatsu Photonics), W-VIEW GEMINI image splitting optics (Hamamatsu Photonics), and AT-X M100 PRO D macro lens (Tokina) for the recording. Although C8600-05 GaAsP image intensifier unit was used for other experiments, including the recordings from a primary culture of hippocampal neurons and freely-moving mice, C9546-02 GaAsP high-speed-gated image intensifier unit (Hamamatsu Photonics) equipped with a gate function was used to protect the image intensifier unit from the large current evoked by the visual stimulation light. Bioluminescence emitted from samples during imaging was split by a FF509-FDi01-25×36 dichroic mirror (Semrock) and passed through emission filters (NLuc channel; FF02-472/30-25 and FF01-483/32-25, Venus channel; FF01-537/26-25 and FF01-542/27-25) in W-VIEW GEMINI image splitting optics (Hamamatsu Photonics). During the recording from head-fixed mice, the mouse head was held using the attached headplate, and the mouse was placed over the custom-made running disk (**Fig. 2E**), which was attached to the rotary encoder to record the running speed of the mouse under the control of LabView (National Instruments). The cranial window was placed around the center of the field of view and the camera binning was set to 4. The distance between the lens and the mouse was 100 mm and the area of FOV was 9.8×19.6 mm. The distance between the tip of the lens and the mouse, and the field of view (FOV) could be adjusted by selecting a lens suitable for the experimental purpose (**Fig. 2E**).

For visual stimulation (light illumination to the right eye) during the bioluminescence imaging (from left V1 neurons in awake mice), a 15, 30, or 45-mW blue laser from Sapphire 458 LP (Coherent) was coupled to 3-mm-diameter liquid light guide (Thorlabs) through F35 plano-convex lens (Sigma), chopper wheel (Thorlabs), F40 plano-convex lens (Sigma), iris diaphragm (Sigma), and 12.7-mm-aperture optical shutter (Thorlabs), as shown in **Supplementary Fig. 3A**. A thin white tape covered the tip end of the liquid light guide to sufficiently scatter the output light and protect the mouse

retina. The output power density of the 15, 30, and 45-mW blue laser was 0.55, 1.10, and 1.65 mW/cm^2, respectively, at the surface level of the eye (there was a 1-cm distance between the eye and tip end of the liquid light guide).

During this experiment, a mouse head was fixed under the camera lens by holding the attached headplate as described above. Three different light intensities were sequentially tested for each mouse without changing the positions of the mouse head and tip of the optical fiber. Light pulses (2-ms duration for a single pulse, with a 23-ms interval (40 Hz)) generated by a chopper wheel were delivered for 1 s, with an inter-trial interval of 7 s, using the optical shutter (**Supplementary Fig. 3B**). A total of 15 stimulus trials were repeated at each light intensity. Imaging was performed at 40 Hz, and the exposure time was set to 20 ms so that images were captured only when the light illumination was off (called "dead-time imaging")[14,23]. Therefore, a light-driven artifact was not present in the detected signals. Exposure and gate-on timing were controlled using a TTL signal from a multifunction generator (WF1973, NF Corporation), with the output signal from the optical chopper system acting as the trigger.

***In vivo* imaging of freely moving mice.** During *in vivo* imaging, the freely moving mice were placed in the cage and signals were detected using the same equipment used for imaging head-fixed mice, except that wide-angle lens (HF12.5SA-1, Fujinon), C8600-05 GaAsP image intensifier unit (Hamamatsu Photonics) and no emission filters were used. The camera binning was set at 2 and 8 to record multiple mice and a single mouse, respectively. The frame rate was set at 10–100 Hz (they were adjusted according to the expression level and brightness of the bioluminescence to maintain a high signal-to-background ratio (SBR) and clearly identify the signal from the movie). The distance between the lens and the mice was 415–572 mm and the area of FOV was 132×264–170×340 mm. The distance between the tip of the lens and the mouse, and the field of view (FOV) could be adjusted by selecting the lens suitable for the experimental purpose (**Fig. 3A**).

The merged movies shown in **Supplementary Videos 1 and 3** were performed to confirm that the signal was correctly detected from the cranial window (**Supplementary Video 1**) or to capture the shapes of mice and target brain areas during imaging from multiple mice (**Supplementary Video 3**). These movies were produced using bright-field images acquired under a 632-nm Light Emitted Diode (LED) (LightEngine SPECTRA, Lumencor) illumination, and bioluminescence images acquired in the dark, which were captured alternately every 50 ms, as previously described [6,8]. The field of view was illuminated by LED with an irradiation time of 5 ms from the initiation of the camera exposure, once every two frames; the timing was controlled with a TTL signal from a multifunction generator (WF1973, NF Corporation).

The TTL signal was generated using the output signal from the exposure time-out signals from an EMCCD camera as the trigger.

Data analysis. All imaging data were processed using Fiji, R-software (Version 3.2.2.) and MATLAB (Mathworks). The curve fitting was performed using Origin 8.5.1 (OriginLab).

Neuronal activity was measured by calculating the FRET ratio (R) of LOTUS-V (Venus signal divided by NLuc signal) as previously described [9]. The background signal was subtracted from the signal of the specimen before the FRET ratio was calculated. The method of background subtraction depended on each imaging setup. For the cultured hippocampal neurons and head-fixed mouse imaging, the "background signal" in both NLuc and Venus channels, measured in a randomly chosen region of interest (ROI) and placed at a non-specimen area, was subtracted from the signal of the specimen in each channel. When the freely moving mice were imaged, the "background image," that was made by median stack from 1000 blank images taken with the closed shutter of an EMCCD camera, was subtracted from the acquired images. The change in the FRET ratio ($\Delta R/R_0$) was calculated by subtracting the average value in the baseline (R_0) from individual raw values at each time-point ($\Delta R = Rt - R_0$; Rt is a raw value at time-point "t") and further dividing the difference, ΔR, by the R_0. The meaning of the term "baseline" also depended on the experiment or analysis. During *in vitro* experiments in cultured hippocampal neurons, the term "baseline" meant that neurons were at the resting membrane voltage. The term "baseline" for locomotion analysis, however, meant that the mice were immobile and not moving (resting state, <5 cm/s for head-fixed mice, <1 cm/s for tracking data from freely-moving mice; the threshold of each was set based on the level of noise fluctuation of the detected the velocity when mice were actually not moving, and by referring to a previous study [10]). The term "baseline" for the interaction analysis meant that a mouse was distant enough from others (distant state, >4 cm) or in the "distant and resting" state, while for the visual stimulation trials, it meant that a mouse was in the resting state during the absence of stimulation.

Each single imaging frame acquired via the W-VIEW GEMINI image splitting optics (Hamamatsu Photonics) contained information from two channels (Venus and NLuc signals), at either the left or right side. To align the two-channel data during post-processing, we first acquired non-bioluminescence images under white light so that the shape of the neuron (*in vitro*), cranial window (head-fixed mice), or cage (freely moving mice) could be similarly observed in each channel. These reference frames were separated to each channel. Then, using the coordinate information, the two channels of the

actual imaging movies were aligned so that we could calculate the LOTUS-V FRET ratio (*R*) using common ROIs. During the *in vitro* and head-fix experiments, drifting or motion-based shift of the sample was rarely observed. However, to confirm that the ROI could correctly work in all frames of each movie, we performed motion correction as previously described [5].

When we analyzed movies of primary culture of hippocampal neurons, the ROI was created from a mask image, which was made based on an averaged picture over all frames of each movie (with single-channel data). The threshold for bioluminescence intensity was manually determined to cover the expression area (e.g., top 0.5%; however, we obtained similar results using a variety of thresholds). The following Boltzmann function was used to fit the voltage-sensitive curve in primary hippocampal neuronal cultures:

$$\frac{\Delta R}{R_0} = \frac{C_1}{1 + \exp(\frac{Ze(V - V_{1/2})}{kT})} + C_2$$

where, C_1 and C_2 were constant values, e was the elementary electric charge, Z was the effective valence, k was the Boltzmann constant, T was the room temperature in K, and $V_{1/2}$ was the voltage at which $\Delta R/R_0$ is half-activated.

To analyze voltage kinetics, we fitted the activation and deactivation curves of $\Delta R/R_0$ using the following two-component exponential equation:

$$\frac{\Delta R}{R_0} = C_3 \exp(\frac{t - t_0}{\tau_1}) + C_4 \exp(\frac{t - t_0}{\tau_2}) + C_5$$

where, C_3, C_4, and C_5 were constant values (C_3 and C_4 were used to calculate the fraction of τ_{fast}), t_0 was the initial time-point, and τ_1 and τ_2 were the time constants for the fast and slow components.

The protocol used for *in vitro* experiments were also used to create the ROIs when analyzing the data from the movies of head-fixed mice. The relationship between the LOTUS-V signal and mouse locomotion was analyzed as described above.

When imaging the freely moving single-housed mice, the bioluminescent spot derived from the cranial window in the field of view was automatically tracked using the “Particle Track Analysis” (PTA ver 1.2; https://github.com/arayoshipta/projectPTAj) as described in **Supplementary Fig. 4**. When analyzing data of interactively moving mice, the cranial window in the headplate seen in the bright-field image was manually tracked using “Manual Tracking” (ImageJ plugin). The trajectories shown in **Figs. 3B, 4A, Supplementary Videos 2 and 4,** were pseudo-colored, based on the velocity or z-normalized $\Delta R/R_0$.

Since we used a relatively high gain of the image intensifier (4-5 out of 10 levels) to decrease the exposure time and increase the frame rate, the dark noise observed as bright spots apart from the bioluminescent signal easily contaminated the images, which was often misrecognized as the signal by PTA for automatic tracking analysis. Thus, we separately performed the "tracking step" and "intensity measurement step" (**Supplementary Fig. 4).** For the tracking part, we used the filtered images in which the effect of dark noise was minimized with a temporal filter (**Supplementary Fig. 4**, "Tracking"). In contrast, the non-filtered images were used for precise intensity measurement of the bioluminescent spots, based on the coordinate information obtained with automatic tracking analysis (**Supplementary Fig. 4**, "Intensity measurement"). Based on the fact that the dark noise rarely appeared at the same pixels in two consecutive frames, the bioluminescence and blank images were processed using the 1D temporal median filter (which is equivalent to a 3D median filter whose (x,y,t) radii are equal to (0,0,1); median filtering at every 3 frames) to reduce the noise (**Supplementary Figs. 4A and B**). In addition, to correct the inhomogeneous background level caused by the camera itself, a background image (described as "blank images" in **Supplementary Fig. 4**) was made by more than 1000 blank images acquired when the camera's shutter was closed. This background image was subtracted from the filtered bioluminescence images (**Supplementary Figs. 4B and C**). The gaussian blur filter was then applied with a radius value set to 1.0 to further decrease the noise and make the identification of the actual brain signal easier in the PTA (**Supplementary Fig. 4D**). The filtered bioluminescence images were separated into NLuc and Venus channels and aligned with each other, by referring to the bright-field image taken before the experiments to visualize and register the position of the mouse cage in each channel (**Supplementary Fig. 4E**). The separated and aligned channels were merged using the "OR" function of Image J to make more reliable reference images (**Supplementary Fig. 4F**). By tracking the bright spot in the eventual images using PTA, we obtained the coordinate information (**Supplementary Fig. 4G**). Since the signal intensity at each channel (not the calculated FRET ratio) was affected by the angle of the mouse head (as shown in **Supplementary Fig. 6**), some of the frames were difficult to automatically track due to the low SBR. Therefore, when the coordinate information could not be automatically detected in more than 3 frames using PTA, these missed frames were excluded from the analysis. If the signal appeared within 3 frames (i.e., with two frames missed at most) from the position where the last signal was detected, then the PTA recognized them as a series, placed the ROI at their position, and continued automatic tracking (in these cases, linear interpolation was used to fill the missing signal values).

For the further "intensity measurement" step, the bioluminescence images of NLuc and Venus channels were prepared again from the same movie "without" the process of 1D temporal median filter (**Supplementary Figs. 4H-J**). The intensities from the bioluminescent spots in both channels at each time point were measured by locating ROIs based on the coordinate information obtained in the previous step (**Supplementary Fig. 4K**). When measuring the signal intensity (i.e., averaged pixel values within the ROI), 32 bit images offered more unbiased intensity measurement than 16 bit images, especially if the bioluminescent signal intensity was low and more easily affected by photon shot noise. This is because 32 bit images allow the negative pixel values that can contain intensity information lower than the value of the background image (i.e., the averaged image from more than 1,000 blank images), during the step of background subtraction (**Supplementary Fig. 4H**) [11]. In some frames, the signal intensity obtained by locating the ROI was slightly smaller than 0, which is also often observed in a single-molecule analysis [11]. Since these negative values can disperse the distribution of the ratio value (Venus intensity divided by NLuc intensity), the absolute minimum value through a series of the imaging sessions (e.g., several movies taken for a specific mouse) was added to the all values in both the NLuc and Venus channels, respectively. If the signal intensity in the NLuc or Venus channel was 0, the frame at that time point was removed before data analysis. Finally, we calculated the Venus/NLuc ratio values (**Supplementary Fig. 4L**). The velocity of the mouse was also calculated from the frame-by-frame position changes at the center of the gravity of the bioluminescence signal derived from the mouse brains.

For the manual tracking analysis, we tracked the position of the cranial window in the headplate by referring the bright-field images. After manually detecting the coordinate information, we measured NLuc and Venus signals as we did for the automatic tracking analysis and calculated the ratio values.

Dark noise during imaging was main and strong source of artifacts (0~0.8), which was sometimes much stronger than the actual signals (>2.0), and often overlapped with the bioluminescence signal in the image. To minimize the effect of the dark noise, ratios within the 0.8–2.0 range was selectively used for data analysis. This range was carefully set by referring to the result of the head-fixed mouse (the averaged ratio value plus or minus threefold the value of standard deviation, N=8 mice).

The SBR was calculated by dividing the averaged intensity of NLuc and Venus emissions by the background intensity. Automatic tracking could not properly distinguish the bioluminescence signal from dark noise when the SBR was lower than 0.12 (due to substrate consumption and/or an original low-expression level of LOTUS-V). Thus, low SBR data

were excluded from the analysis. The total imaging period for each mouse was estimated by the duration of high SBR (0.12 or higher).

When imaging multiple mice, 1 mouse (of the 4 mice) was relatively immobile and did not voluntarily interact with others (**Fig. 4, Supplementary Videos 3 and 4**). To quantitatively evaluate how immobile each mouse was, the fraction of the active state (>1 cm/s) [10] was calculated. Although the average fraction of the mice used for the free-moving experiments was 64.2±7.8% [mean ± SE.] (N=11 mice), that of the quiet one (N=1) was only 2.1%. Following justification of the normal distribution of the data (Kolmogorov–Smirnov test, $p > 0.05$, N=12 mice), a “Chi-squared test for outliers” systematically detected this quiet mouse as an outlier ($p < 0.05$). Therefore, the data of this mouse was excluded from the analysis.

To statistically compare results obtained from different mice expressing either LOTUS-V or LOTUS-V(D129R), the $\Delta R/R_0$ in each mouse was z-normalized [12] to obtain “z-normalized $\Delta R/R_0$”. The z-score values were calculated by subtracting the average baseline signals from individual raw values, and further dividing the difference by the baseline standard deviation. When we analyzed the data during visual stimulation, first 0.5 s of data was used as the signals during visual stimulation, while baseline (resting state) signals were calculated by randomly choosing the same number of frames (with that of data during visual stimulation) from whole resting state data of each mouse for respective comparisons (i.e., 10,000 repeats of random choices were used to calculate the representative (average) value for each “mouse,” at each light intensity; or one time random selection to obtain a group of baseline signals for each “trial” at each light intensity).

When analyzing the activity of V1 during the interaction of multiple mice, the area and the position of each mouse in the 2D image were also approximated using three circles (**Supplementary Fig. 10A**), by referring to a previous study [13]. The positions of the nose (x_1, y_1), headplate (x_2, y_2), and tail root (x_3, y_3) were manually tracked. This coordinate information was further used to calculate the positions of the cervix ($2x_2 - x_1$, $2y_2 - y_1$) and dorsum (($x_3 + 2x_2 - x_1$)/2, ($y_3 + 2y_2 - y_1$)/2). The centers of three variable circles were then set at the headplate (Circle 1), cervix (Circle 2), and dorsum (Circle 3), respectively. The radius of each circle was set as the distance between the headplate and nose (for Circle 1 and Circle 2), or that between the dorsum and tail root (for Circle 3). Thereafter, the distances between the nose of the target mouse and edge of circles of other mice were calculated (**Supplementary Fig. 10B**). The shortest one was used as a “nose-to-body distance” for the interaction analysis (**Supplementary Figs. 10B and C**). When the nose was the inside of the circle of other mice, the distance was represented as a negative value.

Since LOTUS-V reported an increase of V1 activity by visual stimulation (**Supplementary Fig. 3C**), one might be concerned that bioluminescence from other mice could work as visual input. To exclude this possibility, we evaluated the power density of LOTUS-V bioluminescence from the V1 surface. The number of photons, *P*, collected on an EMCCD camera sensor was calculated from the total analogue-to-digital converter (ADC) counts, *I*, using the following equation:

$$P = \frac{IA_{\mathrm{d}}}{Qt_{\mathrm{ex}}G},$$

where, A_{d} was the analog to digital conversion factor of an EMCCD camera, *Q* was the quantum efficiency of an EMCCD camera, t_{ex} was the exposure time, and *G* was the radiant emittance gain of an image intensifier unit. Since the ray divergence, θ from the bioluminescent object was small enough, the fraction of the collected photons, *F*, was calculated using the following equation:

$$F = \frac{\pi (W_{\mathrm{d}} \tan\theta)^2}{2\pi W_{\mathrm{d}}^{\;2}},$$

where W_{d} was the working distance of a lens.

Therefore, the bioluminescence intensity, *B*, radially emitted from the V1 surface was calculated using the following equation:

$$B = \frac{hcP}{\lambda F},$$

where, *h* was the Planck constant, *c* was the speed of light; and λ was the wavelength. Accordingly, the power density of LOTUS-V bioluminescence from V1 surface was computed to be $(7.6 \pm 1.0) \times 10^{-9}$ and $(9.0 \pm 1.3) \times 10^{-9}$ mW/cm^2 (N=6 mice), at 480 nm and 540 nm, respectively. Since the weakest visual stimulation (0.55 mW/cm^2) did not influence the LOTUS-V signal (**Supplementary Fig. 3C**), the increase of V1 activity during the interaction was unlikely to be caused by the bioluminescence from other mice (**Figs. 4C and D**).

Supplemental References

1. Roy, A., Kucukural, A. & Zhang, Y. I-TASSER: a unified platform for automated protein structure and function prediction. *Nat. Protoc.* **5,** 725–738 (2010).
2. Zhao, Y. *et al.* Microfluidic cell sorter-aided directed evolution of a protein-based calcium ion indicator with an inverted fluorescent response. *Integr. Biol. (Camb).* **6,** 714–25 (2014).
3. Inutsuka, A. *et al.* Concurrent and robust regulation of feeding behaviors and metabolism by orexin neurons. *Neuropharmacology* **85,** 451–460 (2014).

4. Grimm, D. *et al.* In vitro and in vivo gene therapy vector evolution via multispecies interbreeding and retargeting of adeno-associated viruses. *J. Virol.* **82,** 5887–5911 (2008).
5. Agetsuma, M., Hamm, J. P., Tao, K., Fujisawa, S. & Yuste, R. Parvalbumin-Positive Interneurons Regulate Neuronal Ensembles in Visual Cortex. *Cereb. Cortex* **28(5),** 1831–1845 (2018).
6. Saito, K. *et al.* Luminescent proteins for high-speed single-cell and whole-body imaging. *Nat. Commun.* **3,** 1262 (2012).
7. Goldey, G. J. *et al.* Removable cranial windows for long-term imaging in awake mice. *Nat. Protoc.* **9,** 2515–2538 (2014).
8. Matsushita, J. *et al.* Fluorescence and Bioluminescence Imaging of Angiogenesis in Flk1-Nano-lantern Transgenic Mice. *Sci. Rep.* **7,** 46597 (2017).
9. Inagaki, S. *et al.* Genetically encoded bioluminescent voltage indicator for multi-purpose use in wide range of bioimaging. *Sci. Rep.* **7:42398,** (2017).
10. Saleem, A. B., Ayaz, A., Jeffery, K. J., Harris, K. D. & Carandini, M. Integration of visual motion and locomotion in mouse visual cortex. *Nat. Neurosci.* **16,** 1864–1869 (2013).
11. Wang, Y. *et al.* Single molecule FRET reveals pore size and opening mechanism of a mechano-sensitive ion channel. *Elife* **3,** e01834 (2014).
12. Herry, C. *et al.* Switching on and off fear by distinct neuronal circuits. *Nature* **454,** 600–6 (2008).
13. de Chaumont, F. *et al.* Computerized video analysis of social interactions in mice. *Nat. Methods* **9,** 410–417 (2012).
